# Aspirin to target arterial events in chronic kidney disease (ATTACK): study protocol for a multicentre, prospective, randomised, open-label, blinded endpoint, parallel group trial of low-dose aspirin vs. standard care for the primary prevention of cardiovascular disease in people with chronic kidney disease

**DOI:** 10.1186/s13063-022-06132-z

**Published:** 2022-04-21

**Authors:** Hugh Gallagher, Jennifer Dumbleton, Tom Maishman, Amy Whitehead, Michael V. Moore, Ahmet Fuat, David Fitzmaurice, Robert A. Henderson, Joanne Lord, Kathryn E. Griffith, Paul Stevens, Maarten W. Taal, Diane Stevenson, Simon D. Fraser, Mark Lown, Christopher J. Hawkey, Paul J. Roderick

**Affiliations:** 1 SW Thames Renal Unit, Epsom and St Helier University Hospitals NHS Trust, Epsom, UK; 2 Faculty of Medicine and Health Sciences, University of Nottingham, Nottingham, UK; 3 Southampton Clinical Trials Unit, University of Southampton, Southampton, UK; 4 Department of Primary Care and Population Sciences, Faculty of Medicine, University of Southampton, Southampton, UK; 5 School of Medicine, Pharmacy and Health, Durham University, Durham, UK; 6 Carmel Medical Practice, Nunnery Lane, Darlington, UK; 7 University of Warwick, Coventry, CV4 7AL UK; 8 Trent Cardiac Centre, Nottingham University Hospitals NHS Trust, Nottingham, UK; 9 Health Technology Assessment Centre, Faculty of Medicine, University of Southampton, Southampton, UK; 10 York, UK; 11 Kent Kidney Care Centre, East Kent Hospitals University Foundation Trust, Canterbury, UK; 12 School of Medicine, University of Nottingham, Nottingham, UK; 13 University Hospitals of Derby and Burton NHS Foundation Trust, Derby, UK

**Keywords:** Chronic kidney disease, Cardiovascular disease, Aspirin, Primary care, Primary prevention

## Abstract

**Background:**

Chronic kidney disease (CKD) is a very common long-term condition and powerful risk factor for cardiovascular disease (CVD).

Low-dose aspirin is of proven benefit in the secondary prevention of myocardial infarction (MI) and stroke in people with pre-existing CVD. However, in people without CVD, the rates of MI and stroke are much lower, and the benefits of aspirin in the primary prevention of CVD are largely balanced by an increased risk of bleeding.

People with CKD are at greatly increased risk of CVD and so the absolute benefits of aspirin are likely to be greater than in lower-risk groups, even if the relative benefits are the same. Post hoc evidence suggests the relative benefits may be greater in the CKD population but the risk of bleeding may also be higher. A definitive study of aspirin for primary prevention in this high-risk group, recommended by the National Institute for Health and Care Excellence (NICE) in 2014, has never been conducted. The question has global significance given the rising burden of CKD worldwide and the low cost of aspirin.

**Methods:**

ATTACK is a pragmatic multicentre, prospective, randomised, open-label, blinded endpoint adjudication superiority trial of aspirin 75 mg daily vs. standard care for the primary prevention of CVD in 25,210 people aged 18 years and over with CKD recruited from UK Primary Care.

Participants aged 18 years and over with CKD (GFR category G1-G4) will be identified in Primary Care and followed up using routinely collected data and annual questionnaires for an average of 5 years. The primary outcome is the time to first major vascular event (composite of non-fatal MI, non-fatal stroke and cardiovascular death [excluding confirmed intracranial haemorrhage and other fatal cardiovascular haemorrhage]). Deaths from other causes (including fatal bleeding) will be treated as competing events. The study will continue until 1827 major vascular events have occurred. The principal safety outcome is major intracranial and extracranial bleeding; this is hypothesised to be increased in those randomised to take aspirin. The key consideration is then whether and to what extent the benefits of aspirin from the expected reduction in CVD events exceed the risks of major bleeding.

**Discussion:**

This will be the first definitive trial of aspirin for primary CVD prevention in CKD patients. The research will be of great interest to clinicians, guideline groups and policy-makers, in the UK and globally, particularly given the high and rising prevalence of CKD that is driven by population ageing and epidemics of obesity and diabetes. The low cost of aspirin means that a positive result would be of relevance to low- and middle-income countries and the impact in the developed world less diluted by any inequalities in health care access.

**Trial registration:**

ISRCTN: ISRCTN40920200. EudraCT: 2018-000644-26. ClinicalTrials.gov: NCT03796156

**Supplementary Information:**

The online version contains supplementary material available at 10.1186/s13063-022-06132-z.

## Introduction

### Background

Chronic kidney disease (CKD) is defined as any abnormality of kidney function or structure with implications for health that is present for more than 3 months. It is classified according to the estimated glomerular filtration rate (eGFR) and urine albumin:creatinine ratio (UACR). The presence of an eGFR < 60 mL/min/1.73 m^2^ or an UACR ≥3 mg/mmol for more than 90 days is diagnostic of CKD.

CKD is common, particularly in older people. The prevalence of CKD is estimated at 12–13% of adults from population data in England [1] and the USA [2]. An important minority of people with CKD will develop end-stage kidney disease (ESKD), but the greatest significance of CKD is as a powerful and potentially modifiable risk factor for cardiovascular disease (CVD). People with CKD are categorised according to Kidney Disease Improving Global Outcomes (KDIGO) classification as being at moderate risk, high risk or very high risk of CVD according to the level of both eGFR and UACR [3]. In the USA, 9.2%, 2% and 0.8% of adults are in the moderate risk, high risk and very high risk categories [4]; these proportions were similar in the Health Survey of England [5].

Large-scale robust epidemiological data indicate that the risks of both all-cause and cardiovascular mortality in the general population increase where the eGFR is less than 60 mL/min/1.73 m^2^, and/or where the UACR is greater than 1 mg/mmol. The risks are graded: compared with eGFR 95 mL/min/1.73 m^2^, adjusted hazard ratios (HR) for all-cause mortality were 1.18 (95% CI = 1.05–1.32) for eGFR 60 mL/min/1.73 m^2^, 1.57 (1.39–1.78) for 45 mL/min/1.73 m^2^ and 3.14 (2.39–4.13) for 15 mL/min/1.73m^2^. UACR was associated with risk of mortality linearly on the log-log scale without threshold effects. Compared with UACR 0.6 mg/mmol, adjusted HR for all-cause mortality were 1.20 (1.15–1.26) for UACR 1.1 mg/mmol, 1.63 (1.50–1.77) for 3.4 mg/mmol, and 2.22 (1.97–2.51) for 33.9 mg/mmol. eGFR and UACR were multiplicatively associated with risk of mortality without evidence of interaction. Similar findings were recorded for cardiovascular mortality [6].

Albuminuria and eGFR are similarly predictive of mortality, independent of traditional cardiovascular risk factors, in high-risk population cohorts [7] and kidney disease cohorts [8], and in people with and without diabetes [9] and hypertension [10]. These findings hold true in older people [11], both sexes [12] and across ethnic groups [13].

In people without previous myocardial infarction (MI), the rate of MI is higher in those with CKD (without diabetes) than in those with diabetes (without CKD): 6.9 per 1000 person-years (6.6–7·2) vs. 5.4 per 1000 person-years (95% CI 5.2–5.7) ; *p* < 0.0001) [14]. In the Finnish Diabetic Nephropathy study, excess mortality in people with type 1 diabetes was only observed in those with CKD [15]. In the Third National Health and Nutrition Examination Survey, those with kidney disease were found to predominantly account for the increased mortality observed in type 2 diabetes [16].

The pattern of vascular events in people with CKD varies according to disease severity. For those with the most severe impairment in GFR, and in particular those with ESKD receiving renal replacement therapy, atherothrombotic events are less prevalent and arrhythmia and heart failure more important [17]. However, in those where the GFR is less severely impaired, and where albuminuria indicates the presence of vascular damage and endothelial dysfunction [18], atherothrombotic events dominate.

The burden of CVD in CKD is substantial. The risk of a cardiovascular events in people with CKD is far higher than the risk of ESKD [19]. Overall, CVD is responsible for about one-third of all deaths in the UK. It can have a serious impact upon quality of life and cause considerable disability. CKD is included as a vascular condition within the English Department of Health’s CVD Outcomes Strategy [20]. The financial impact of CVD in CKD is large: assuming unit costs of £12,200 for a stroke and £7734 for an MI and incidence of stroke and MI of 12.0 and 11.9 per 1000 patient-years respectively in people with CKD [21], the annual costs of strokes and MI in people with CKD in England were estimated in 2009/10 to be in the order of £1bn, greater than the cost of dialysis provision.

Our understanding of how to reduce cardiovascular risk in CKD is limited. The Study of Heart and Renal Protection (SHARP) demonstrated that primary prevention with simvastatin and ezetimibe reduced major atherosclerotic events in people with CKD. CVD event rates were high: 13.4% of a control group (mean eGFR of 27 mL/min/1.73 m^2^) experienced a major atherosclerotic event (including revascularisation) in SHARP over a median follow-up of 4.9 years [22]. Even in a lower-risk UK primary care cohort (mean eGFR 52 mL/min/1.73 m^2^, 84% without albuminuria), 35% experienced a hospital admission with a cardiovascular event over a mean of 5.1 years of follow-up [23] and the annual mortality from CVD in those without pre-existing CVD was as high as 0.7% [24]. Evidence on additional approaches to prevent CVD in CKD is therefore urgently required.

### Rationale

In patients with CVD, there is good evidence that antiplatelet therapy reduces the risk of subsequent vascular events (secondary prevention) and that overall these benefits outweigh the risks of major bleeding, which is the principal complication of therapy. A meta-analysis conducted by the Antithrombotic Trialists’ Collaboration (ATC) showed that antiplatelet agents (primarily aspirin) reduced serious vascular events by 22% across five major high risk categories of patients (previous MI, acute MI, previous stroke or transient ischaemic attack (TIA), acute stroke and other high risk). In 195 trials, there were 7705/71,912 (10.7%) serious vascular events in the antiplatelet-treated group against 9502/72,139 (13.2%) in adjusted controls. There was increased risk of major bleeding: 95/47,158 fatal and 440/47,158 non-fatal major extracranial bleeds (1.1%) were seen in the antiplatelet group against 71 and 62/47,168 (0.7%) in the controls [25]. Antiplatelet therapy is recommended internationally for the secondary prevention of cardiovascular events in people with established cardiovascular disease.

In lower-risk populations without pre-existing CVD, the absolute benefits of aspirin for the primary prevention of CVD are smaller and offset by an increased risk of bleeding. An ATC meta-analysis of six primary prevention studies reported a 12% proportional reduction in serious vascular events in a lower risk population (0.51% vs. 0.57% per annum) with aspirin [26].

Three more recent aspirin primary prevention studies were published in 2018. ASCEND [27] and ARRIVE [28] explored the use of aspirin for primary prevention of CVD in people with diabetes and at moderate CV risk respectively. ASPREE [29] examined the effect of aspirin on disability-free survival in healthy elderly subjects; cardiovascular disease was a pre-specified secondary outcome [30]. The results of these three trials were incorporated into an updated systematic review. This review reported similar findings to previous meta-analyses: a total of 13 trials randomising 164,225 participants with 1,050,511 participant-years of follow-up were included. The median age was 62 years and the median estimated 10-year cardiovascular event rate was 9.2%. Aspirin use was associated with significant reductions in the composite cardiovascular outcome compared with no aspirin (571 per 100,000 participant-years with aspirin and 614 per 100,000 participant-years with no aspirin; hazard ratio [HR] 0.89 [95% credible interval 0.84–0.95]; absolute risk reduction 0.38% [0.20–0.55%]; number needed to treat 265). Aspirin use was associated with an increased risk of major bleeding events compared with no aspirin (231 per 100,000 participant-years with aspirin and 164 per 100,000 participant-years with no aspirin; HR 1.43 [1.30–1.56]; absolute risk increase 0.47% [0.34–0.62%]; number needed to harm 210). For patients at higher risk of CVD (estimated 10-year risk > 10%), the magnitude of both the benefits (absolute risk reduction in composite CV outcome of 0.53%) and harms (absolute risk increase in major bleeding of 0.64%) was higher than in lower risk populations [31].

Despite this substantial body of evidence, uncertainties still remain over whether and under what circumstances aspirin should be used for primary prevention. Whilst the effects on CV events and bleeding appear balanced across the populations studied, determination of the real-world net impact is not straightforward. The definitions of major bleeding employed have been inconsistent, and weighing the overall risks and benefits, and in particular the relative importance of CV and bleeding events, is challenging:

- Fatal bleeding events are uncommon overall. A 2016 meta-analysis found that although aspirin increased the risk of gastrointestinal (GI) bleeding by 60% there was no increase in fatal bleeds [32], although far higher rates of fatal or disabling bleeding in the elderly have been reported in an unselected high risk secondary prevention cohort (including some taking dual antiplatelet therapy) [33].
- It may be possible to mitigate the bleeding risk, but data on gastroprotection have not been reported consistently in the primary prevention literature. Proton pump inhibitors (PPI) reduce the risk of peptic ulcer in at-risk individuals treated with low-dose aspirin by approximately 80% [34]. There is also evidence that H2 antagonists reduce low-dose aspirin-associated bleeding in high risk users [35].
- Fatal CV events are more common. The 30-day day mortality in the survivors of a first MI is around 5% [36]. UK national data for 2010 indicate a 30-day case fatality rate for MI of 31% overall and 12% in those admitted to hospital [37]. However, in the general population, aspirin does not appear to reduce overall CV mortality [31, 38].
- Aspirin may reduce the risk of certain cancers [39, 40].
- The use of aspirin has been associated with a small relative risk reduction in all-cause mortality in meta-analyses published before 2018 [38, 41] but not in the most recent analysis [31].

The effect of aspirin on the highest risk primary prevention populations is also unclear. CKD substantially increases the risks of CV events, with a graded relationship between advancing stages of CKD and rates of fatal and non-fatal MI, and the probability of death following MI [42]. The absolute benefits of aspirin may therefore be higher than in lower-risk populations even if the relative risk reductions are similar. People with CKD have not been well-represented in historical or recent primary prevention studies: the proportion with an eGFR < 60 mL/min/1.73 m^2^ in ASCEND and ASPREE was 12% [43] and 19% [44] respectively. A recent post hoc analysis of people with CKD in ASPREE did not confirm a net benefit of aspirin in elderly people with CKD but was not powered to definitively assess the presence of treatment heterogeneity [45].

It is not clear to what extent any benefits may be offset because people with CKD are also at increased risk of bleeding. Many people with CKD are elderly, which is a risk factor for aspirin-associated bleeding [33]. There are additional specific mechanisms through which the bleeding tendency may be increased in CKD, including defective platelet adhesion to the sub-endothelium, defective platelet aggregation, and other intrinsic platelet defects [46]. A Cochrane review (which included patients at all stages of CKD, including those receiving renal replacement) reported that the use of antiplatelet agents in people with CKD conferred an increased relative risk of major (27 studies, RR 1.33, 95% CI 1.10–1.65) and minor bleeding (18 studies, 1.49 (1.12–1.97)) compared with placebo/control. The definitions of bleeding employed within the included studies were variable. The relative risks of major bleeding due to aspirin appeared no higher than those in the non-CKD population, although the absolute excess risks were higher due to the higher risks in the CKD control groups [47].

The role for gastroprotective agents in people with CKD treated with low-dose aspirin is undefined. Whilst people with CKD may be at higher bleeding risk, an increased incidence of acute interstitial nephritis in users of PPI has been reported. The absolute risks are small, with a nationwide nested case-control study revealing an incidence of 12.0 (95% CI 9.1–15.5) and 1.7 (0.9–1.9) per 100,000 patient-years in current and past users respectively. Observational data have also revealed associations between PPI and incident CKD [48] and of adverse chronic renal outcomes (decline in eGFR of more than 30% and ESKD) in those without intervening acute kidney injury [49]. Whether such pharmaco-epidemiological data should be used to imply a causal link has been recently challenged [50]. H2 antagonists may be an alternative.

Support for a role for aspirin in primary prevention in CKD can be found in data from the Hypertension Optimal Treatment (HOT) trial, which suggest that the relative risk reductions in CVD with aspirin may be greater in people with CKD than in those with normal kidney function. In the overall HOT study population, aspirin reduced the risk of major cardiovascular events by 15%, but did not affect total mortality or cardiovascular mortality [51]. However, there was evidence of significant heterogeneity by eGFR. Major cardiovascular events were reduced by 9% (95% CI − 9 to 24%), 15% (− 17 to 39%), and 66% (33 to 83%) for patients with baseline eGFR of ≥60, 45 to 59, and < 45 mL/min/1.73 m^2^ respectively (*p* for trend = 0.03). In those with an eGFR of 45–59 mL/min/1.73 m^2^, 800 (− 700 to 2200) major cardiovascular events were prevented per 100,000 patients treated for 3.8 years, at a cost of 400 (− 200 to 1000) major bleeds; at eGFR< 45 mL/min/1.73 m^2^, 7600 (3100 to 12,100) events were prevented, at a cost of 270 (− 100 to 5500) bleeds. Total mortality was not affected in the CKD group as a whole but was significantly reduced in those subjects with eGFR < 45 mL/min/1.73 m^2^. On sensitivity analysis, eGFR appeared to define the threshold for benefit. However, this was a post hoc analysis and only 2.9% of the HOT population had an eGFR < 45 mL/min/1.73 m^2^. Reporting of bleeding episodes was imprecise [52]. It is also unclear how generalisable the findings are to normotensive people with CKD as the criteria for entry into HOT were BP-based [51].

A 2016 systematic review exploring the use of aspirin for primary prevention in CKD patients specifically identified three trials from a total of 1314 records screened; two of these provided previously unpublished data. In total, 4468 adults with pre-end-stage CKD and no history of CVD were included. There were 16,740 person-years of follow-up. The trials were assessed as showing medium to high levels of risk of bias, largely related to endpoint assessment and suboptimal identification of CKD. Only one trial, HARP [53], was CKD-specific; it did not report cardiovascular events in aspirin and placebo groups. There was no pre-specified CKD analysis in the other two studies, HOT and the Japanese Primary Prevention of Atherosclerosis with Aspirin for Diabetes (JPAD) trial [54]. Overall, there was no statistically significant reduction in major cardiovascular events (RR 0.92, 95% CI 0.49–1.73, *p* = 0.79). There was a high level of heterogeneity (*I*^2^ = 71% *p* = 0.06). In HOT, there were 76/1791 cardiovascular events in the aspirin-treated group and 110/1828 in controls, with a risk ratio of 0.71 (0.53–0.94). The numbers were smaller and the findings divergent in JPAD, with 24/342 and 15/290 events in aspirin and control groups respectively and a risk ratio of 1.36 (0.73–2.54). Overall, there were 100/2241 CVD events in aspirin-treated patients across the included studies and 125/2228 in controls. Mortality was non-significantly reduced in the aspirin group (RR 0.74, 0.55–1.00, *p* = 0.05, *I*^2^ 0%). Aspirin increased the risk of major bleeding (34/2241 episodes aspirin-treated patients vs. 17/2228 in controls (RR 1.98, 1.11–3.52, *p* = 0.02, *I*^2^ 0%)).

The authors of the systematic review concluded that the limitations of the evidence highlighted the need for definitive CKD-specific randomised controlled trials [55], reiterating the 2014 recommendation of NICE for a definitive trial of aspirin for primary prevention of CVD in people with CKD [56]. This paper outlines the design of such a study.

### Objectives

#### Primary objective

The primary objective of the ATTACK study is to test the hypothesis that low-dose (75 mg non-enteric coated) aspirin reduces the risk of major vascular events (excluding confirmed intracranial haemorrhage and other fatal cardiovascular haemorrhage) in people with CKD who do not have pre-existing CVD.

#### Secondary objectives

The secondary objectives of the research are:

(i) To assess the impact of the addition of low-dose aspirin to usual care in people with CKD and no CVD on the incidence of major intracranial and extracranial bleeds; this is hypothesised to be increased in those randomised to take aspirin. The key consideration in then whether the benefits of aspirin from the expected reduction in CVD events (primary objective) exceed the expected risks of major bleeding;
(ii) To assess the impact of the addition of low-dose aspirin to usual care on other secondary and tertiary endpoints (all-cause mortality, combined endpoint of major vascular events and revascularisation [coronary and non-coronary], individual components of the primary endpoint, TIA, unplanned hospitalisation, hospitalisation for heart failure, new diagnosis of cancer [colorectal/other], death due to cancer [where cancer is the underlying cause of death], major non-traumatic lower limb amputation, CKD progression, health-related quality of life [HRQoL] and dementia);
(iii) To examine a priori the effect of low-dose aspirin on primary, secondary and tertiary endpoints in various subgroups of people with CKD (high-risk and very high-risk CKD as defined by KDIGO on the basis of eGFR and UACR category), diabetes, age ≥ 70, eGFR < 45 mL/min/1.73 m^2^, UACR ≥3 mg/mmol, UACR > 30 mg/mmol);
(iv) To assess the impact of the addition of low-dose aspirin to usual care in people with CKD and no CVD on the incidence of inpatient clinically relevant bleeds not meeting major bleeding criteria;
(v) To assess the cost-utility of low-dose aspirin compared with usual care.

### Trial design

ATTACK is a pragmatic multicentre, prospective, randomised, open-label, blinded endpoint, parallel group, superiority trial of aspirin (75 mg daily non-enteric coated or dispersible) versus no additional treatment (and avoidance of aspirin) in people aged 18 years and over with CKD and no CVD.

The adoption of an open-label over a placebo-controlled design offers the advantage of substantially lower trial costs. Assessment of safety is a critical issue. It is not possible in an open trial to fully mitigate the risk that allocation to aspirin will increase the reporting of symptoms. However, the impact of knowledge of treatment allocation on outcome measurement will be minimised with blinded independent outcome adjudication of major clinical endpoints, including all bleeding events that require hospitalisation.

**Fig. 1:**
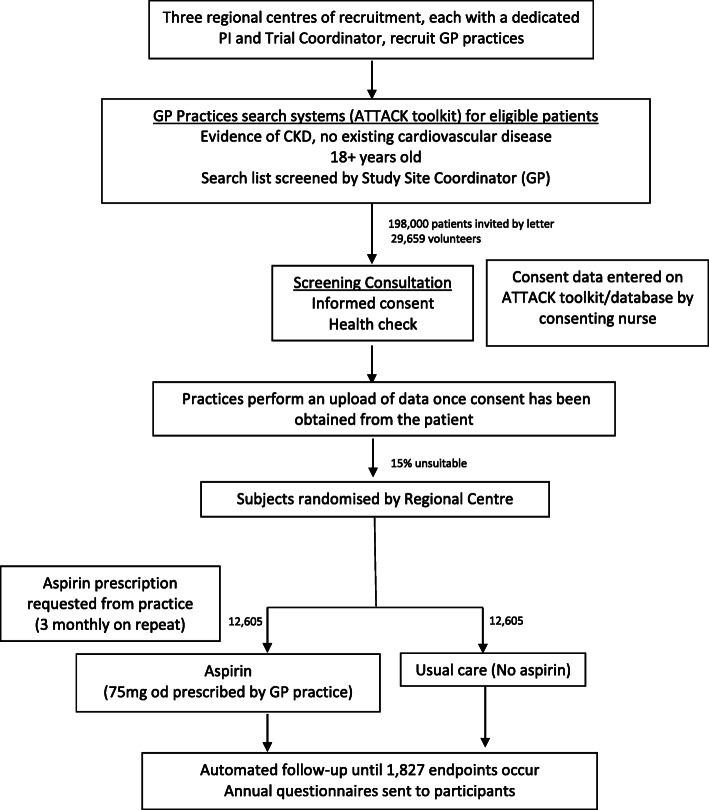
Trial flow diagram. In total 25,210 patients with CKD will be randomised to receive aspirin in addition to their usual medication or no additional treatment (and avoidance of aspirin), and followed up until 1827 adjudicated major cardiovascular events (primary outcome) have occurred. It is anticipated that 3.5 years of recruitment and 2.5 years of follow-up will be required to complete the trial

The ATTACK Trial Flow Diagram is provided in Fig. 1 (Additional files 1 and 5).

## Methods: participants, interventions and outcomes

### Study setting

Participants will be recruited from UK Primary Care (General Practitioner (GP) practices). This is where most people with CKD are treated in the UK, and so this approach should maximise generalisability of the trial results. A list of study sites will be available on request.

### Eligibility criteria

These are summarised in Tables 1 and 2.

**Table 1:** Inclusion criteria

• Males and females aged 18 years and over at the date of screening • Subjects with CKD (reduced eGFR and/or albuminuria) defined as: o Estimated glomerular filtration rate [eGFR] < 60 mL/min/1.73 m^2^ for at least 90 days, and/or o Kidney disease code on the GP electronic patient AND most recent eGFR in CKD-defining range (< 60 mL/min/1.73 m^2^), and/or o Albuminuria or proteinuria (defined as urine albumin:creatinine ratio [ACR] inmg/mmol, and/or urine protein:creatinine ratio [PCR] inine mmol, and/or + protein or greater on reagent strip)* • Subjects willing to give permission for their paper and electronic medical records to be accessed and abstracted by trial investigators for the duration of the trial • Subjects willing to be contacted and interviewed by trial investigators should the need arise for adverse event assessment • Subjects able to communicate well with the investigator or designee, to understand and comply with the requirements of the study and to understand and sign the written informed consent * where albuminuria measurements are not available KDIGO state that measurements of urine protein:creatinine ratio or urine protein reagent strips can be substituted. Negative to trace on protein reagent strip is equivalent to ACR < 3 mg/mmol; trace to + is equivalent to ACR 3–30 mg/mmol [3]. The relationship between reagent strip measures and ACR depends upon urine concentration, and in this context for the purposes of ATTACK, we are regarding +protein or more as indicative of significant albuminuria. A single abnormal albuminuria/proteinuria test is required for entry to the trial: day-to-day variation in albumin excretion is substantial and the literature linking albuminuria to adverse outcomes is predicated upon single ACR readings; robust cohort data confirm that for urine ACR down to 1.7 mg/mmol multiple urine samples do not improve performance of CV mortality risk models beyond information achievable by implementation of one ACR value [57]

**Table 2:** Exclusion criteria

• CKD GFR category 5 by KDIGO classification (eGFR < 15 mL/min/1.73 m^2^) • Pre-existing CVD: angina, MI, stroke (ischaemic or haemorrhagic [intracerebral/subarachnoid]), TIA, significant peripheral vascular disease, coronary or peripheral revascularisation for atherosclerotic disease; aortic aneurysm is not an exclusion criterion • Pre-existing condition associated with increased risk of bleeding other than CKD: upper GI bleed or peptic ulcer in the previous 5 years, lower GI bleed in previous 12 months, active chronic liver disease (such as cirrhosis), bleeding diathesis (investigator opinion) • Taking over the counter aspirin continuously • Currently prescribed anticoagulant or antiplatelet agent • Currently and regularly taking other drugs with a potentially serious interaction with low-dose aspirin, including non-steroidal anti-inflammatories (except topical preparations) and nicorandil • Known allergy to aspirin or definite previous clinically important adverse reaction to aspirin • Poorly controlled hypertension (latest recorded systolic blood pressure [BP] ≥180 mmHg and/or diastolic BP ≥105 mmHg) • Other conditions which in the opinion of the GP would preclude prescription of aspirin in routine clinical practice, for example significant anaemia or thrombocytopenia • Pregnant or likely to become pregnant during the study period • Malignancy that is life-threatening or likely to limit prognosis, other life-threatening co-morbidity, or terminal illness • Behaviour or lifestyle that would render subject less likely to comply with study medication (e.g. alcoholism, substance abuse, debilitating psychiatric conditions or inability to provide informed consent) • In prison • Currently participating in another interventional clinical trial or who have taken part in a trial in the last 3 months (Covid-19 vaccine studies are acceptable)	

### Interventions

#### Description of investigational medicinal product

Active treatment will be aspirin (CAS 50-78-2) 75 mg given once daily. Non-enteric-coated tablets or dispersible preparations may be used interchangeably. Aspirin will be prescribed using the standard NHS prescribing system, which is automatically logged in the GP practice electronic system. Standard labelling and packaging will be used.

Aspirin exerts an antiplatelet action through the irreversible inhibition of cyclooxygenase-1. This prevents the generation of prostaglandins, including thromboxane A2, and endothelial prostacyclin. Thromboxane A2 is an inducer of platelet aggregation and prostacyclin an inhibitor of platelet aggregation. As aspirin is less effective at reducing prostacyclin production than thromboxane A2 generation, the net effect favours reduced platelet aggregation and less thrombus formation [58]. In total, 75 mg is the lowest proven effective antiplatelet dose of aspirin [25]. Equivalent doses of the enteric-coated aspirin are not as effective as plain aspirin [59]. No clear clinical benefits in terms of reduction of GI bleeding or ulceration with enteric coating have been demonstrated [60].

There is no placebo; control subjects will receive no additional treatment to their usual medication and be advised to avoid aspirin or aspirin-containing products.

#### Criteria for discontinuing or modifying allocated interventions

Trial participants will be advised to seek advice from their usual treating physician for any condition arising during the course of the study. Treating physicians will be asked to follow their usual practice for the management of dyspeptic symptoms or anaemia.

The individual trial participant aspirin stopping criteria (in participants randomised to receive aspirin) are as follows: diagnosis of a non-traumatic major bleed or other serious adverse reaction; commencement of treatment with warfarin or other antithrombotic or antiplatelet drug (except where combination therapy with aspirin is clinically indicated); or where there is a clinically important reason for a patient to be commenced on any drug with a strong interaction with aspirin. Any participant who experiences an adverse event may be withdrawn from study treatment at the discretion of the Investigator. Randomised patients who commence renal replacement therapy will not be withdrawn from trial treatment unless another indication for this arises.

Treating physicians will be advised to commence participants in the usual care arm on aspirin where an indication arises.

#### Concomitant medications

There are no mandated concomitant or rescue medications. The risk of bleeding in people with CKD is likely to vary with both age and CKD category. Such heterogeneity is not captured by current clinical guidelines. ATTACK is a pragmatic study, and a real-world approach will also be applied to this area of clinical uncertainty. The decision to introduce gastroprotection, and the choice of any gastroprotective agent, is not mandated, but rather will be at the discretion of the treating GP. Our GP training materials will provide the necessary information to support a process of shared decision-making, highlighting factors that are likely to increase the risks of bleeding.

#### Adherence to prescribed treatment

An analysis of aspirin primary prevention trials reported persistence rates (proportions still taking trial medications/not withdrawing from trial treatments) that varied between 50 and 90% over 3 to 5 years [61]**,** with an average persistence across the six studies of 73% at 4.5 years. In ASPREE 63% of participants were taking study medication during the final year of the trial; 4% were taking open-label aspirin in year 5 [62]**.** Incomplete adherence in the aspirin arm will also dilute the treatment effect measured by intention-to-treat (ITT) analysis, reducing the relative risk towards the null. However, the estimated risk reduction in ATTACK is conservative and has been carefully considered in the light of other aspirin trials analysed using ITT. As near-complete routine outcome follow-up data will be available, the threat to internal validity as a result of different withdrawal rates between the two arms will be minimal.

Self-reported compliance with prescribed aspirin and over the counter aspirin consumption in the usual practice arm will be assessed in an annual questionnaire. Treatment adherence will also be assessed from routine GP prescribing data. A regular report will be downloaded to monitor any subjects on the aspirin arm of the trial who have not had an aspirin prescription in the last four months.

Where poor adherence is demonstrated the project team will intervene pro-actively to try and address the issue. Where needed, research staff may attempt to contact patients directly to discuss and emphasise the importance of taking the study medication.

### Outcomes

The definitions of clinical endpoints used in ATTACK are detailed in Appendix 1 (Additional file 4).

#### Primary endpoint

The primary outcome measure is the time to first major vascular event from the date of randomisation through to the end of follow-up. A major vascular event is defined as a primary composite outcome of non-fatal myocardial infarction, non-fatal stroke and cardiovascular death (excluding confirmed intracranial haemorrhage and other fatal cardiovascular haemorrhage). Deaths from other causes (including fatal bleeding) will be treated as competing events. Patients who do not experience a major vascular event will be censored at the date of last follow-up.

#### Secondary endpoints

The secondary endpoints will be analysed as time to event from date of randomisation through to the end of follow-up with the exception of health-related quality of life (HRQoL). HRQoL will be measured using annual EQ-5D-5L questionnaires [63] and converted to utilities. Cost utility analysis will be performed to determine incremental costs and health improvements expressed in the unit of quality adjusted life years.

(i) Efficacy

- Death from any cause
- Composite outcome of major vascular event or revascularisation (coronary and non-coronary)
- Individual components of the primary composite endpoint
- Health-related quality of life

(ii) Safety

- Composite outcome of intracranial haemorrhage (fatal and non-fatal), fatal extracranial haemorrhage and non-fatal major extracranial haemorrhage (adjudicated)
- Fatal and non-fatal (reported individually and as a composite) intracranial haemorrhage comprising:
  - Primary haemorrhagic stroke (to distinguish from haemorrhagic transformation of ischaemic stroke): (i) intracerebral and (ii) subarachnoid haemorrhage (reported individually and a composite) (adjudicated)
  - Other intracranial haemorrhage: (i) subdural and (ii) extradural haemorrhage (reported as a composite) (adjudicated)
  - Intracranial haemorrhage will be subcategorised as traumatic or non-traumatic [64]
- Fatal and non-fatal (reported individually and as a composite) major extracranial haemorrhage: (i) upper gastrointestinal; (ii) lower gastrointestinal; (iii) sight-threatening ocular; (iv) multiple trauma; (v) other (adjudicated)
- Clinically relevant non-major bleeding (if hospitalised) (adjudicated)
- Composite outcome of fatal and non-fatal major extracranial haemorrhage and clinically relevant non-major bleeding (if hospitalised)

#### Tertiary endpoints

Exploratory endpoints will be analysed as time to event from the date of randomisation through to the end of follow-up except hospitalisations, which will be reported as a rate over time.

- Transient ischaemic attack
- Unplanned (emergency) hospitalisations
- Hospitalisation with heart failure
- New diagnosis of cancer (colorectal/other)
- Death due to cancer (where cancer is the underlying cause of death)
- CKD progression (at least one of: > 30% fall in eGFR over 2 years [65]; need for renal replacement therapy or 50% decline in eGFR [66]; new eGFR< 15 mL/min/1.73 m^2^; 25% decline in GFR together with a drop in GFR category [3])
- New diagnosis of dementia
- Major non-traumatic lower limb amputation

### Recruitment

#### Recruitment system

There will be three geographical recruitment hubs based at Regional Centres in Southampton (South), Nottingham/Warwick (Midlands) and South Tees (North). Each hub will be supported by a dedicated Trial Coordinator and Principal Investigator (PI). The activities of the hubs will be coordinated and monitored by the Trial Manager based at the University of Nottingham.

GPs will identify potentially eligible patients at their practice using an automated search. The practice will be able to download the ATTACK toolkit required to perform the search from the web. The toolkit will contain query files to perform searches on the GP practice clinical system based on the inclusion and exclusion criteria. The automated searches use a combination of biochemical test results and coded clinical terms. The Read coded prevalence of CKD GFR categories 3 to 5 in England is 4.1% of people aged 18 years and over [67]. This is substantially lower than the estimated actual prevalence of 6.1% of people aged 16 and over [68]**.** Unlike CKD G3-5, the coding of CKD GFR categories 1–2 has never been incentivised under the Quality and Outcomes Framework (QOF) and is therefore likely to be far less complete than that for CKD G3-5. Miscoding of CKD is also common: 11% of people with a CKD 3-5 Read code in the National CKD Audit did not have current biochemical evidence of CKD [69]. For these reasons, both numerical values for eGFR and albuminuria/proteinuria and clinical terms will be used to identify potential participants.

The search will return a list of potential patients which will be held within the practice. The GPs will check the list of patients to confirm potential eligibility and indicate that patients can be contacted and screened by signing the search list and documenting any exclusions.

An automated invitation pack will be sent to the eligible patients via Docmail, a highly secure online mail management system. The pack will include a participant invitation letter, a copy of the Research Ethics Committee (REC)-approved Participant Information Sheet (PIS) and informed consent form (ICF), a reply slip and pre-paid return envelope (addressed to the Regional Centre).

#### Feasibility

Test searches at practices participating in the *Helicobacter* Eradication Aspirin Trial (HEAT) [70], indicated an average of 370 potentially eligible patients per practice. A rate of randomisation of 15% would give 55 participants per practice. With a more pessimistic set of assumptions, the trial remains feasible. The prevalence of CKD 1–5 is in the order of 12% from population data. The National Diabetes Audit highlighted that there are over 1 million people with diabetes and CKD 1–2 [71]. Not all of these patients will have blood and urine tests that are diagnostic of CKD on their GP records. If 8% of adults can be diagnosed with CKD 1–5 on the basis of test results, and of these 70% have no pre-existing CVD, and 80% of these are not taking aspirin, then a typical practice could potentially enrol around 300 eligible patients. A number of these will be excluded on other grounds (for example taking prohibited concomitant medications). If the rate of randomisation is 8%, full recruitment will be possible from the network of 1200 practices participating in HEAT (1257 enrolled, 1163 active [96% in England]) [72], with whom the ATTACK investigators have existing links through a common trial management team. If the number of eligible patients and/or the consent rate was lower, still there is nonetheless the ability to recruit additional practices outside the HEAT network: overall 48% of general practices across England take part in National Institute for Health Research (NIHR) Clinical Research Network (CRN) Portfolio studies [73]. As in HEAT, there is also scope to extend into Scotland, Wales and Northern Ireland.

### Participant timeline

#### Summary schedule of enrolment, interventions and assessment

**Fig. 2:**
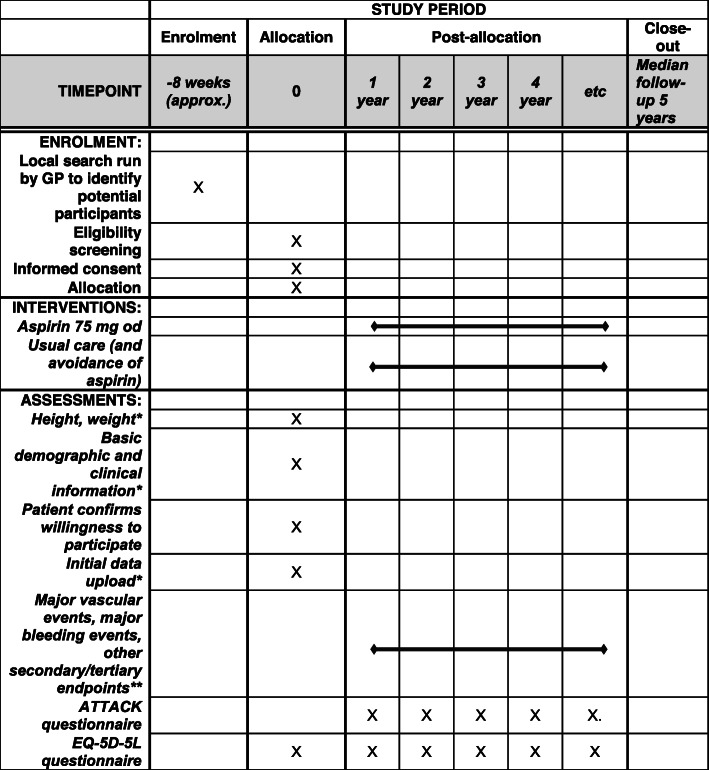
Schedule of enrolment, interventions and assessment. *Extracted from GP EPR. **Combination of: linkage to HES/ONS data, extraction from GP EPR, participant self-reporting, and reporting by GP and hospitals

The schedule of enrolment, interventions and assessment is provided in Fig. 2.

#### Consent consultation

Potential participants who respond to express an interest will be contacted, primarily by telephone, to give them further information and allow them to ask questions. Suitable patients will be invited to a consent consultation.

In the light of the 2020 Coronavirus pandemic, a decision was taken in Summer 2020 to offer remote consultations. No screening tests will now be taken; instead, laboratory-based checks of eligibility will be based on pre-existing blood and urine test results available within the GP electronic patient record (EPR). The effect of this is to render the trial entirely Covid-secure, as no face-to-face assessment are required at any stage in the trial. This is important as CKD is a risk factor for poor outcomes in Covid-19 [74]. Prior to this, potentially eligible patients in ATTACK underwent screening eGFR and UACR testing at a face-to-face screening visit to confirm eligibility.

During the virtual consent consultation, inclusion and exclusion criteria will be checked and the patient consented by an appropriately trained research nurse or registered medical professional with suitable study training. If the consenting individual has any concerns over the eligibility of a patient, they will discuss it with the GP at the practice, who will ultimately decide if the patient is suitable.

Latest blood pressure will be extracted from the EPR. Basic demographic and clinical details will be recorded at consent, including self-defined ethnicity, height and weight, smoking history, and alcohol consumption. All participants will also complete an EQ-5D-5L questionnaire.

Additional information, including postcode (used to generate Index of Multiple Deprivation), summary diagnoses, cardiovascular risk factors (for example diabetes [type and duration], hypertension and lipid profile) and concomitant medications will also be automatically extracted from the EPR as required.

The GFR category at entry will be determined according to the most recent CKD-EPI eGFR, currently corrected for ethnicity. For sites where the Modification of Diet in Renal Disease (MDRD) eGFR is reported, CKD-EPI eGFR will be calculated from the standardised serum creatinine. The inclusion of correction factors for ethnicity has been reassessed by the American Society of Nephrology and National Kidney Foundation [75], and we have adopted the recommendations from NICE in August 2021 to remove these [76].

The exclusion of potential participants on the grounds of bleeding diathesis or anaemia is on the basis of investigator opinion. Thrombocytopenia is an important indicator of diathesis, but the risk of bleeding at any given platelet count is likely to be related to many factors including age, blood pressure, kidney function and, in the case of gastrointestinal bleeding, the presence of *Helicobacter pylori* infection. There are no widely accepted protocols governing the use of aspirin in thrombocytopenia [77], and very limited evidence to guide decision-making. It has been argued that aspirin can probably be safely continued in patients post cardiac bypass surgery with platelet counts below 50 × 10^9^/L, unless clinical bleeding occurs or the count falls below 20 × 10^9^/L [78]; others have recommended (“in the absence of evidence”) stopping antiplatelet agents in people with stable coronary artery disease and a platelet count < 50 × 10^9^/L [79]. We have therefore not specified a fixed platelet count below which participants are automatically ineligible, but where thrombocytopenia (platelets < 70 × 10^9^/L) is present on the latest blood test (within the previous two years), this will be flagged at the Regional Centre for GP review. We will ask the Data Monitoring and Ethics Committee (DMEC) to review bleeding risk subdivided by platelet count. Where the latest haemoglobin (within the previous two years) is < 90 g/L (or < 100 g/L with mean cell volume (MCV) ≤75 fL), this will also be highlighted to the potential participant’s GP. Patients will be excluded if, based on a holistic assessment of their bleeding risk, the GP would be unwilling to prescribe aspirin for them (should an indication arise) outside the trial. By applying usual practice, the results of ATTACK should have the greatest applicability to real-world clinical medicine. Where no FBC is available within the last 2 years, the latest available results will be marked for GP review, following the same principle.

#### Randomisation

Randomisation will take place only once all consent paperwork has been received at the Regional Centre from the participant and the consenting nurse.

#### Follow-up assessments

There is no practice-based follow-up. Potential outcomes will be determined from a combination of routinely collected healthcare (GP and hospital) data, including death certification and cancer registration; reporting by GPs and admitting hospitals; and self-reporting by patients. All events identified as a potential cardiovascular endpoint or bleeding event requiring hospital admission will be formally adjudicated; outpatient sight-threatening eye bleeds will also be adjudicated.

The Regional Centres will regularly download a report from the ATTACK database to monitor if any trial participant does not have an eGFR reading recorded in their GP record in the previous 15 months. If this is the case, the GP Practice will be contacted to remind them of the NICE guideline to perform these tests annually as a minimum [56].

### Sample size

A total of 25,210 patients (12,605 per arm) will be required in order for the required 1827 major vascular events to be observed.

#### Initial sample size estimate (not accounting for competing risks)

An initial sample size was calculated using NQuery v4.0 assuming a 2% annual usual care event rate and powered to detect a HR of 0.868 for the risk of experiencing a major vascular event with aspirin (proportion event-free at 5 years: 90.4% (usual care) vs. 91.6% (aspirin)). With 85% power, 5% two-sided alpha, 3.5 years for recruitment, 2.5 years follow-up and 1% dropout (withdrawal of consent for follow-up), a total of 1792 major vascular events would be required overall.

#### Definitive sample size estimate (accounting for competing risks)

As the primary outcome measure involves competing risks (deaths from other causes, including deaths from fatal bleeding which are anticipated to be higher in the aspirin arm), a sample size adjustment calculated using the Cumulative Incidence approach is required as recommended by Pintilie and Tai [80, 81]. Methods to calculate the sample size in the presence of competing risks [80, 81] were used under the following assumptions:

- Proportional hazards assumption holds between the two arms
- A 2% annual major vascular event rate in the usual care arm
- An initial HR of 0.868 (equivalent to a 1.74% annual major vascular event rate in the aspirin arm)
- A 1.8% annual event rate in the usual care arm for deaths from other causes (including fatal bleeding)
- A 1.85% annual event rate in the aspirin arm for deaths from other causes (including fatal bleeding), i.e. assuming that patients in the aspirin arm will experience a 0.05% annual rate increase of fatal bleeding compared to patients in the usual care arm
- 85% power, 5% two-sided alpha, 1% dropout rate
- 3.5-year recruitment period and 2.5-year follow-up period

The corresponding sample size information was calculated as follows (all values rounded to 4 decimal places):

- Cumulative incidence at 5 years (in the presence of competing risks) for the usual care arm of 0.0911
- Cumulative incidence at 5 years (in the presence of competing risks) for the aspirin arm of 0.0796
- Subdistribution HR of 0.8692
- Proportion of main event failures in the usual care arm of 0.0782
- Proportion of main event failures in the aspirin arm of 0.0682
- Pooled proportion of main event failures of 0.0732
- Number of major vascular events required of 1827
- Number of patients required (prior to an allowance of dropout) of 24,958
- Number of patients required (after an allowance of dropout) of 25,210 (12,605 per arm)

#### Estimation of effect size

An initial HR of 0.868 (12.5% RR reduction at five years) is both clinically important and appropriate for the ATTACK study population. This estimate is based upon current knowledge on the use of aspirin for primary and secondary prevention [26, 31], the risk profile of people with CKD [82] and the results observed in the subgroup of participants in the HOT study who had CKD [52].

#### Estimation of vascular event rate

It is not possible to predict the control event rate for this trial with certainty. The largest cardiovascular outcome trial in CKD was the Study of Heart and Renal Protection (SHARP), where the annual rate of major cardiovascular events (non-fatal MI, non-fatal stroke and CV death) was 1.8% in the control group [22]. Compared with the SHARP population (mean age 62, mean eGFR 27 mL/min/1.73 m^2^), the ATTACK participants are likely to be older but have less impaired renal function. This is important: age is a powerful predictor of vascular events, and risk is also related to CKD severity; the net effect of these two opposing factors upon the event rate in ATTACK is not yet clear.

The observed rate of major vascular events in a given trial population is however likely to be lower now than it would have been 10–20 years ago. More contemporary CKD cohorts also offer important insights. The annual cardiovascular mortality in those without pre-existing CVD in the contemporary Renal Risk in Derby (RRID) primary care cohort was 0.77% [24], implying an annual event rate of 2.3% assuming a ratio of 1.8:1 of non-fatal:fatal cardiovascular events [22]. In the RRID cohort overall (mean eGFR 52 mL/min/1.73 m^2^, 16% with albuminuria, 22% with pre-existing CVD), the annual rate of fatal and non-non-fatal CVD was 2.5% [83]. In the Alberta CKD cohort, the rate of coronary death or non-fatal MI (i.e. excluding stroke) was 1.3% in an older (age ≥ 50 years) but lower risk CKD population without either diabetes or pre-existing coronary heart disease [84].

ATTACK is a pragmatic study and the estimated event rate of 2% assumes that the trial participants will be rather more representative of the real-world CKD population than a very highly selected group of younger and fitter patients that one might expect to see in a more demanding placebo-controlled study involving multiple visits and additional tests.

As the event rate will be highly dependent upon the age and CKD severity of patients recruited, the age distribution and CKD stage of participants will be closely monitored during the first phase of the pilot in advance of the formal estimation of the control event rate, which will take place during the second phase. This will allow time to titrate the number of practices according to the recruitment rate per practice and top up our practice numbers in anticipation of a lower event rate, and to focus recruitment on more severe CKD, thereby enriching the ATTACK population with people at higher risk, should the trial population be younger than expected.

A report to the DMEC on adjudicated major CVD events and bleeding events (by arm) is planned for 45 months into the study based on 27 months of actual recruitment (estimated 23% of primary endpoint events, 226 in the control arm). Advice will be sought from the Trial Steering Committee (TSC) should the event rate differ significantly from that anticipated**.** The time points for this review may change if there are major contextual events that influence trial recruitment and/or progress.

## Methods: assignment of interventions

### Allocation

Consenting patients will be randomised (open label randomisation) 1:1 via an independent web-based system (TENALEA) using random-block size, to GP prescription of aspirin vs no additional treatment (and avoidance of aspirin), stratified by age, diabetes and CKD severity. The randomisation system is run by the ALEA^TM^ team in Southampton and monitored and checked by the Southampton Clinical Trials Unit (CTU). The Regional Centres will enrol participants and assign to the intervention.

### Blinding

Patients and study staff will be aware of the randomisation decision, as there is no blinding to treatment allocation. Outcome adjudicators will be blinded to treatment allocation. The DMEC will see unblinded data for the purposes of assessing risks and benefits. Trial investigators will be unblinded for the assessment of severity and causality of any reported adverse events.

## Methods: data collection, management and analysis

### Data collection methods

#### Data sources

Potential outcomes will be ascertained from four data sources: Office for National Statistics (ONS) for mortality and cancer registration; Hospital Episode Statistics (HES) for hospital admissions; general practice EPR for coded CVD episodes, bleeding episodes, coded diagnoses of dementia, recorded eGFR, and prescription of aspirin and other relevant medications; and healthcare professional- or self-reported information. Self-reported information will include that from an ATTACK patient questionnaire (which will collect data on adherence and outcome events) and an EQ-5D-5L questionnaire. Patients will be asked to complete these follow-up questionnaires annually, either on-line, or by their preferred method of contact (paper/electronic). If required, reminders may be sent.

Clinical outcomes will be classified according to standard frameworks (International Classification of Disease [ICD]-10 disease codes and Office of Population Censuses and Surveys [OPCS]-4 procedure codes) linked to structured clinical vocabularies/dictionaries of clinical terms (SNOMED CT, Read version 2 and Read version 3 [CTV3] [85]).

#### Endpoint adjudication

The sources of outcome data will be cross-referenced in order to build up a potential event record. Potential CVD and major bleeding events will be formally adjudicated. Notification of a potential study endpoint will trigger the collection and redaction of information for endpoint confirmation and blinded adjudication by the Endpoint Adjudication Committee (EAC).

There will be separate adjudication committees for coronary heart disease, cerebrovascular and major bleeding endpoints. The Chairs of the EAC will be responsible for operationalising the definitions of outcome events to ensure application by the committee members that is both feasible and consistent. A consensus adjudication model will be followed, whereby two reviewers discuss the cases and reach agreement. Where agreement is not reached, the case will be discussed with the Chair to determine the final adjudicated outcome. The adjudication process will run in parallel to systems for safety assessment.

#### Participant retention

The use of routinely collected hospital, GP and national mortality and cancer data will allow a full ITT analysis on all participants who are randomised, including those who discontinue study treatment as a result of a clinical decision, non-compliance with the Protocol or drug toxicity, with the exception of those who withdraw from the study. Withdrawal is defined as the withdrawal of consent for record linkage and the collection of follow-up data. We are assuming a 1% dropout rate according to this definition as we are expecting almost complete linkage of the trial participants to national data on hospitalisation (HES), deaths and new cancers (ONS) which will enable event capture and adjudication. Subjects will be free to withdraw from the trial at any time and will be informed that should they withdraw data collected prior to withdrawal may be used in the final analysis if they agree at this time. Subjects will be contacted annually and thanked for their valuable contribution to the study.

Subjects who do not participate in annual follow-up for EQ-5D-5L and self-reported health events and health service contacts will still be followed up for major outcomes.

### Data management

#### Data forms and data entry

Data recorded by the research nurse at the screening consultation will be entered electronically into the ATTACK database. A source data worksheet will be completed, which will record basic demographic and clinical information about the patient, along with confirmation of inclusion/exclusion criteria. This will be filed in the Trial Master File held at each of the Regional Centres, with a copy also stored in the Site File at each trial practice.

Extracted data relating to Read V2 and V3 codes of relevance from the GP EPR will automatically populate the ATTACK database. GP records will be searched and updated as regularly as the extraction system will allow (this will vary according to the system supplier of the GP EPR). Adaptations to the trial IT architecture in response to changes in the NHS operating environment (for example any transition from Read codes to SNOMED CT in the primary care electronic record) will be performed according to need.

HES and ONS will be accessed annually via NHS Digital. If practices in Wales are required, GP records will be linked to the Patient Episode Database for Wales from the NHS Wales Informatics Services. Record linkage for clinical events in Scotland will be carried out for patients within the trial if needed using national record linkage systems (Information Services Division, NHS National Services Scotland) as in the ALL-HEART trial [86].

Data from the annual ATTACK questionnaire and EQ-5D-5L will be automatically uploaded or electronically entered into the ATTACK database according to need. The results of endpoint adjudication and any serious adverse events will be electronically entered into the ATTACK database.

All data entry will take place either in the Regional Centres or locally where the data originated. The type of activity that an individual may undertake and their level of access to the ATTACK database will be determined by the privileges associated with their log-in details.

#### Data coding

Each participant will be assigned a screening number, and a trial randomisation number, allocated at randomisation, for use on trial documents and the electronic database. The documents and database will also use their initials and date of birth.

#### Status reports

The ATTACK database will produce status reports regularly and on request, including information on recruitment numbers, missing data, aspirin prescription and eGFR testing in trial participants.

#### Data storage and security

Case report forms (CRF) will be treated as confidential documents. The CRF will only collect the minimum required information for the purposes of the trial. CRFs will be held securely, in a locked room, or locked cupboard or cabinet. Access to the information will be limited to the trial staff and investigators and relevant regulatory authorities.

A separate confidential record of the participant’s name, date of birth, local hospital number or NHS number and Participant Trial Number (the Trial Recruitment Log) will be held securely on the trial database, to permit identification of all participants enrolled in the trial in accordance with regulatory requirements and for follow-up as required.

Computer-held data including the trial database will be held securely and password protected. All data will be stored on a secure dedicated web server within the NHS Private Data Network, to which only authorised study personnel will have access. This is compatible with and has the relevant security policies in place, to obtain patient-matched hospital admission data and ONS data for consented patients from NHS Digital. Access will be restricted by user identifiers and passwords (encrypted using AES-25S encryption). Electronic data will be backed up every 24 h to both local and remote media in encrypted format.

#### Data retention

In adherence with the International Conference on Harmonisation (ICH) Good Clinical Practice (GCP) guidelines, the Chief or local Principal Investigator will maintain all records and documents regarding the conduct of the study. These will be retained for up to 10 years after the date of any publication based on the research data. If the responsible Investigator is no longer able to maintain the study records, a second person will be nominated to take over this responsibility. The Trial Master File and trial documents held by the Chief Investigator on behalf of the Sponsor shall be finally archived at secure archive facilities at the Sponsor site. This archive shall include all trial databases and associated metadata encryption codes. This will all be in accordance with the Sponsor’s Standard Operating Procedures (SOP).

### Statistical methods

Analyses will be intention-to-treat (ITT), consisting of all patients who have consented and have been randomised to a treatment arm.

For the primary outcome measure (composite of non-fatal myocardial infarction, non-fatal stroke and cardiovascular death [excluding confirmed intracranial haemorrhage and other fatal cardiovascular haemorrhage]), deaths from other causes (including fatal bleeding) will be treated as competing events. Patients who do not experience a major vascular event will be censored at the date of last follow-up.

As non-fatal major bleeding and anticoagulation are events, which, in the intervention arm, may lead to aspirin cessation, sensitivity analyses of the primary outcome measure (for the ITT population) will also include:

- Censoring patients who experience non-fatal major bleeding (adjudicated), clinically relevant non-major bleeding, or anticoagulation at the date of the event (whichever occurs first)
- Censoring only patients who experience non-fatal major bleeding (adjudicated) at the date of the event

For the secondary outcomes of time to fatal/non-fatal major haemorrhage (both intracranial and extracranial), the following competing risk models will be used to assess impact of assumptions over competing risk and censoring:

- Deaths from other causes (excluding fatal bleeding) will be treated as competing events. Patients who experience a major vascular event will be censored at the date of the event. Patients who do not experience either a major vascular event or fatal/non-fatal major event will be censored at the date of last follow-up
- Major vascular events and deaths from other causes (excluding fatal bleeding) will be treated as competing events. Patients who do not experience a fatal/non-fatal major vascular event will be censored at the date of last follow-up
- Major vascular events and deaths from other causes (excluding fatal bleeding) will be treated as competing events. Patients who experience anticoagulation or clinically relevant non-major bleeding will be censored at the date of the event (whichever occurs first). Patients who do not experience either anticoagulation, clinically relevant non-major bleeding, or a fatal/non-fatal major vascular event will be censored at the date of last follow-up
- Deaths from other causes (excluding fatal bleeding) will be treated as competing events. Patients who experience anticoagulation, clinically relevant non-major bleeding, or a major vascular event will be censored at the date of the event (whichever occurs first). Patients who do not experience either anticoagulation, clinically relevant non-major bleeding, a major vascular event or a fatal/non-fatal major event will be censored at the date of last follow-up

Time to event data will be described using Kaplan-Meier curves (or Cumulative Incidence curves for time to event outcomes involving competing risks). Analyses of time to event outcomes will be performed using a Cox proportional hazards model (or Fine and Gray’s adaptation of the Cox proportional hazards model for the subdistribution of a competing risk [87], i.e. a Competing Risk regression model for time to event outcomes involving competing risks), both unadjusted and adjusted for stratification factors: age, diabetes and CKD severity. The proportional hazards assumption will be assessed graphically with a log-log plot and a Schoenfeld test based on scaled Schoenfeld residuals.

The adjusted competing risk regression model for time to first major vascular event, with deaths from other causes (including fatal bleeding) treated as competing events, and patients who do not experience a major vascular event censored at the date of last follow-up, will form the primary endpoint analysis model.

Negative binomial regression will be used to analyse unplanned hospitalisations, both unadjusted and adjusted for stratification factors: age, diabetes and CKD severity.

For other secondary and tertiary endpoints, we will compare proportions for categorical data and means/medians for continuous data using Pearson’s *χ*^2^ test and *T* test/Mann-Whitney *U* test, respectively.

The amount of missing data and reasons for the incompleteness will be explored and presented overall, i.e. not by group. If the amount of missing data is deemed too high and if appropriate (i.e. assuming the missing data is either missing at random or missing completely at random and censoring assumed to be non-informative), multiple imputation will be performed accordingly, for which all covariates included in the multivariable model, together with the censoring/event indicator and the cumulative baseline hazard will be included in the multiple imputation model.

All statistical analyses will be carried out using Stata v15 or higher, or SAS v9.4 or higher.

### Health economic analysis

Economic analysis will follow the methods and “reference case” recommended by NICE [88]. Modelling will be used to estimate the net effect of aspirin prescribing on healthcare costs and quality-adjusted survival over a lifetime horizon, using trial data to estimate effects on vascular and bleeding risks, cancer incidence, CKD progression and mortality. Trial data will also be used to estimate health-related quality of life and healthcare costs for the population and associated with adverse events.

Costs will be estimated using individual-level linked HES/GP data, supplemented where necessary with information from the patient questionnaire. Costs will be estimated for services potentially affected by aspirin use, including prescriptions (aspirin, gastroprotective and other related drugs); primary care consultations; unplanned admissions for bleeds and vascular events, with related follow-up (e.g. revascularisations); renal replacement therapy following CKD progression.

Unit costs for services will be obtained from standard national sources: NHS Reference Costs for admissions and other hospital services; Personal Social Services Research Unit estimates for primary care and community services; and British National Formulary/Drug Tariff for drug prices.

Quality-adjusted life years (QALYs) will be estimated using data on survival and quality of life (EQ-5D-5L) questionnaires. EQ-5D-5L scores (“utilities”) will be calculated using a UK general population value set, as recommended by NICE at the time of analysis [89, 90]. Costs and QALYs will be discounted at NICE recommended rates (currently 3.5% per year for both).

The model structure, parameter sources and methods of analysis will be specified in a detailed economic protocol paper, informed by a review of high-quality CKD and CVD prevention models. We expect to use an individual-level discrete-event simulation approach to reflect the multiple, competing risks of vascular, haemorrhagic and other related events in this population over a lifetime horizon, taking advantage of the large pragmatic trial dataset [91]. Distributions of baseline characteristics and risk factors will be estimated from trial data. Control arm data will be used to characterise event rates under usual care: e.g. using Cox proportional hazards predictive equations for CVD events and CKD progression; and parametric survival models (e.g. Gompertz) for all-cause survival (pre- and post-event, and by CKD stage or severity) [92]. Relative treatment effects will be taken from the main trial analyses described above under "Statistical methods" (Cox proportional hazards or competing hazards regressions). The impact of events on patients’ quality of life (EQ-5D-5L utility scores) and NHS costs will be estimated from trial data by an appropriate regression approach [93]. If an effect on cancer incidence is found, this will be included in the economic model, although we may need to source background risk, cost and utility parameters for this outcome from the literature.

Uncertainty over model results will be explored through sensitivity analysis. Deterministic analysis will be used to investigate the sensitivity of results to input parameters and key modelling assumptions. Probabilistic analysis will be used to assess the extent and impact of uncertainty over model inputs. Results will be stratified by pre-defined subgroups and CVD risk.

Validity of the model will be assessed by a Health Economist not involved in its development. This will include tests of internal validity: checks that input parameters match specified sources and inspection of coding (white box validation), stress testing of model behaviour (black box validation) and comparison of modelled event rates during the trial follow-up period with trial observations. External validity will be assessed by comparison of intermediate model results (event rates) with relevant estimates from the literature (identified by systematic review).

## Methods: monitoring

### Trial management

The Sponsor of the trial is the University of Southampton. ATTACK is managed from a central Trial Coordinating Centre based at the University of Nottingham. The Southampton CTU will support all statistical processes, including ongoing central statistical monitoring and preparation of open and closed trial reports, randomisation design, set-up and support.

A Trial Steering Committee (TSC) provides overall supervision on behalf of the Sponsor and Funder and ensures that the project is conducted to the rigorous standards set out in the Department of Health’s Research Governance Framework for Health and Social Care and the Guidelines for Good Clinical Practice. The Chair and members have been appointed by the NIHR Health Technology Assessment (HTA) Programme Director according to standard procedures. TSC meetings will have a minimum of 75% majority of independent members, including the Chair. Details of the terms of reference for the TSC are available on request from the ATTACK trial office.

A Data Monitoring and Ethics Committee (DMEC) will monitor unblinded comparative data, supplied in strict confidence, and make recommendations to the TSC on whether there are any ethical or safety reasons why the trial should not continue, ensuring that the safety, rights and well-being of the trial participants are paramount. The DMEC comprises a statistician and two clinicians with expertise in the clinical area. All members are independent and have been appointed by the NIHR HTA Programme Director according to standard procedures. Details of the terms of reference for the DMEC are available on request from the ATTACK trial office.

### Data monitoring

Safety will be closely assessed throughout the trial. The absolute and relative risks of major bleeding will be examined by the DMEC and compared with those expected from the literature. All-cause mortality and the primary event rate will also be studied in order to determine net benefit, i.e. benefits minus harms. Aspirin use requires a consideration of the balance of risk vs. benefit in all populations. The DMEC will recommend termination of the trial if, in their view, the randomised comparisons provided have proven beyond reasonable doubt that the level of harm is unacceptable; or the use of aspirin is clearly contraindicated (or clearly indicated) in terms of the net effects. Clinical judgement will be required in interpreting the results of interim analyses and reaching recommendations. The DMEC will consider whether the evidence meets standards for treatment recommendations and practice guidelines, mindful that less evidence should be required to stop the trial for harm than benefit given the primacy of patient safety [94]. The absolute number of major bleeding events during ATTACK is likely to be low, and therefore, the confidence levels around any estimates of absolute and relative risk will be initially wide but narrow throughout the course of the trial. Hazard ratios may be unstable and drift over time into marginal levels of significance. Multiple “looks” at the data may give rise to a transient “signal” of benefit or harm [95]. Therefore, criteria of proof beyond reasonable doubt cannot be specified precisely, but in general, a difference of at least three standard deviations in an interim analysis of a major endpoint would be needed to justify halting, or modifying, such a study prematurely, especially for a comparison based on relatively few events (< 100) [96].

There are other instances where the DMEC may consider it advisable to advise termination of the study: flaws in design or conduct of the study come to light; or external new information on the treatment becomes available; or resources are inadequate to complete the trial.

### Interim analyses

#### Internal pilot

The first 24 months of the study (9 months set up and 15 months recruitment) are planned as an internal pilot. The key objective of the pilot is to assess GP and patient recruitment. Additional objectives are to finalise major event assessment procedures, monitor safety and assess fidelity to allocated group and patient withdrawal rates. The timings of the pilot period and interim analyses are subject to change if major contextual events impact on trial progress. This was evident in 2020–2021 when recruitment was held as a result of the SARS-Cov-2 pandemic.

##### GP and patient recruitment

Data will be provided on the number of practices overall, and by area, that indicate willingness to take part, perform eligibility assessment and start patient recruitment. The number (and percent per list size) of eligible patients per practice and the number and percentage of eligible patients willing to participate and commencing the trial will be recorded. At the end of the pilot phase, traffic light criteria will be used to establish whether the trial should continue without modification (green); study recruitment strategy changes are required (amber); or the trial should discontinue (red).

##### Endpoint assessment procedures

The adjudication process will be explored and refined during the pilot. Hospital discharge summaries will serve as the primary source of potential endpoint events. These will be assessed and categorised into clear major event or no event, or more information required. In the latter situation, the feasibility, value and costs will be explored of obtaining specific additional information from the original hospitalisation such as ECGs, CT scan results, photocopied medical notes to assess symptoms and post mortem results if in-hospital death. Events that are uncertain will be reassessed using whatever additional information can be obtained.

##### Safety

The plan is to assess safety after 15 months of recruitment (and allowing 3 months for report writing) from any bleeding events requiring hospitalisation in both arms using coded GP data, HES data, healthcare professional-/self-reporting and any other serious adverse event (SAE). This early review will be based on unadjudicated data due to the anticipated delay in receiving HES data. If the DMEC have concerns that fall short of “beyond reasonable doubt” on the basis of the unadjudicated data they will also have the option to halt the trial pending a process of formal adjudication.

##### Fidelity

Fidelity to allocated group by will be studied by examining repeat prescribing data from GP systems and the results of follow-up questionnaires in those reaching 12 months after recruitment.

##### Withdrawal

Withdrawal will be reported as the number (%) of patients who withdraw from the study and refuse access to linked routine data.

#### Subsequent interim analyses

It is anticipated that the DMEC will subsequently review unblinded data: after 24 months recruitment using non-adjudicated data (allowing 3 months for report writing); after 27 months recruitment using adjudicated data (allowing 9 months for adjudication and report writing); and at annual intervals thereafter using adjudicated data, or more frequently if specified by the DMEC.

#### Estimation of event rate

A confidential report to the DMEC on adjudicated major CVD events and bleeding events (by arm) is planned at 45 months into the study based on 27 months of actual recruitment (estimated 23% of primary endpoint events, 226 in the control arm).

Data on adjudicated major CVD events and bleeding events (overall, not by arm) will be reviewed by the TSC at its regular meetings. The TSC will have the option of increasing the sample size or prolonging the scheduled treatment period if the event rate is substantially lower than anticipated.

### Harms

**Fig. 3:**
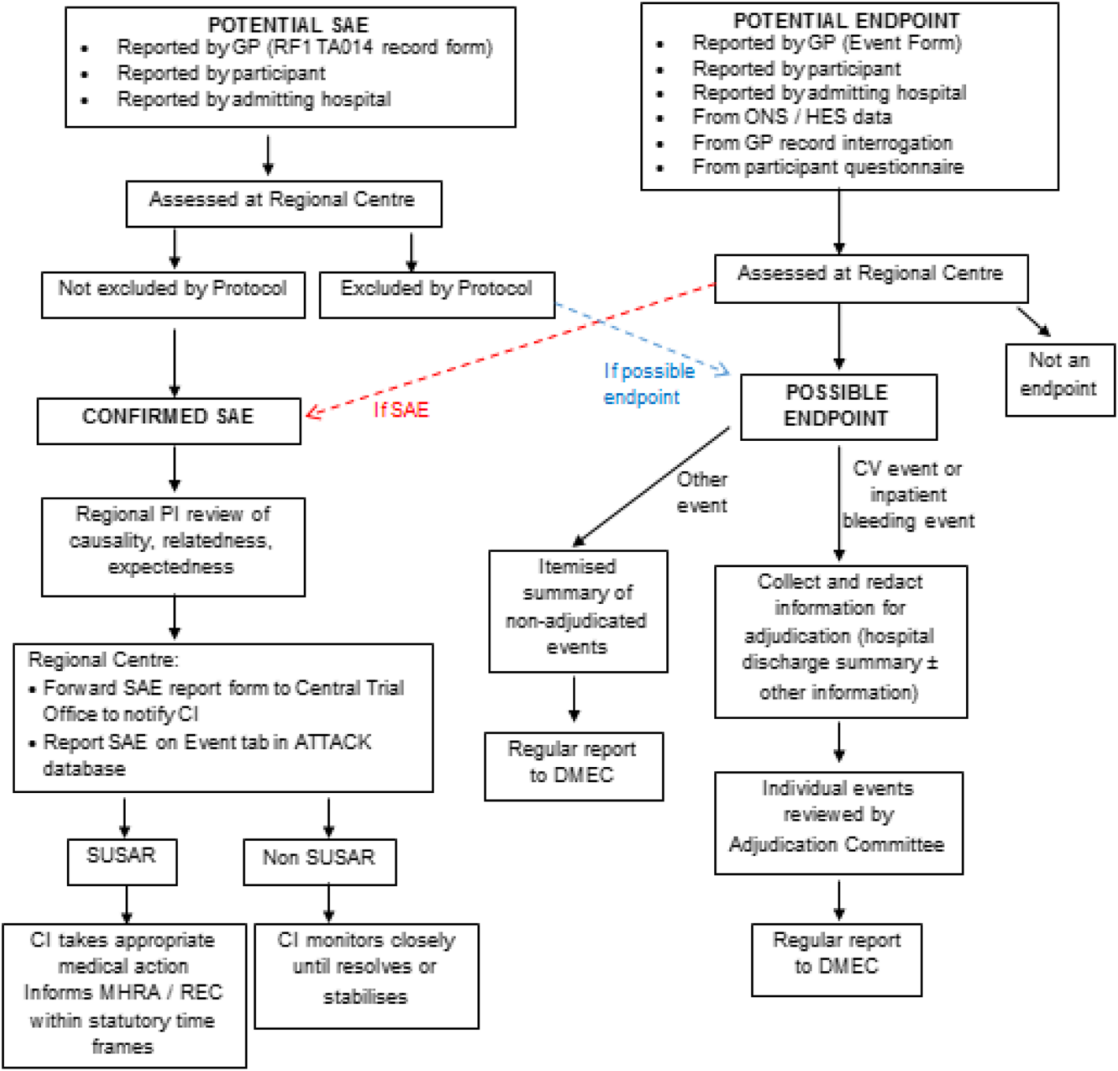
Ascertainment of serious adverse events and trial endpoints

The processes for reporting of SAE and ascertainment of outcome events will work in parallel. This is illustrated in Fig. 3.

The following events are exempted from expedited reporting using an SAE report form:

- Events meeting the definition of SAE but which are listed as undesirable effects in the current Summary of Product Characteristics for aspirin (with the exception of hypersensitivity/allergic reactions which will subject to expedited reporting)
- Anything that constitutes a trial endpoint, as this will be assessed as part of the trial
- SAE which in the opinion of the Investigator are with reasonable probability unrelated to aspirin

Participating GP will be asked to contact the Regional Centres and provide details of potential SAE that are not excluded from expedited reporting as soon as they become aware of the event. Participants will be asked to contact the study site in the event of any emergency hospital admission. They will carry a Trial Participant ID card, which asks admitting hospitals to inform the Regional Centre of hospitalisations. Standard information will be collected and recorded on the CRF by the Regional Centre.

The Regional Centre will screen all potential SAE. Those not excluded will be recorded on an SAE report form. The Regional Principal Investigator will review causality, relatedness and expectedness, and forward the SAE report form to the Trial Coordinating Centre (as soon as possible and within 24 h of becoming aware of the event) who will notify the Chief Investigator. SAE identified in this way will be recorded and closely monitored until resolution and stabilisation or until it has been shown that the study medication or treatment is not the cause. Confirmed reports will be promptly forwarded unblinded to the Chair of the DMEC. All serious adverse events that fall or are suspected to fall within the criteria for a suspected unexpected serious adverse reaction (SUSAR) shall be treated as such until deemed otherwise. The event shall be reported immediately (within 24 h) of knowledge of its occurrence to the Chief Investigator. Safety information relating to adverse events not subject to expedited reporting that are captured as trial endpoints will be closely monitored by the DMEC throughout the trial.

### Auditing

The Regional Centre team, or where required, a nominated designee of the Sponsor, shall carry out monitoring of trial data as an ongoing activity. Monitoring of trial data shall include confirmation of informed consent; source data verification; data storage and data transfer procedures; local quality control checks and procedures; back-up and disaster recovery of any local databases; and validation of data manipulation.

The Trial Coordinating Centre at the University of Nottingham will undertake monitoring of the Regional Centres, focussing on quality assurance, data integrity, adherence to the Protocol and checking training.

The Sponsor will undertake proportionate monitoring of the processes of the Trial Coordinating Centre, Regional Centres and CTU.

Trial data and evidence of monitoring and systems audits will be made available for inspection by the regulatory authority as required.

## Ethics and dissemination

### Funding

ATTACK is jointly funded the National Institute for Health Research Health Technology Assessment Programme (HTA Project: 16/31/127) and the British Heart Foundation (Ref: SP/17/14/33355). The views expressed are those of the author(s) and not necessarily those of the NHS, the NIHR or the Department of Health.

### Trial registration

The trial was prospectively registered in EudraCT (2018-000644-26) on 9th October 2018, ISRCTN (ISRCTN40920200) on 12th October 2018, and at ClinicalTrials.gov (NCT03796156) on 8th January 2019. The trial website is https://www.southampton.ac.uk/attack-trial.

### Protocol amendments

Substantial and non-substantial Protocol amendments will be submitted to the regulatory authorities for approval in accordance with guidance from the HRA. All significant Protocol modifications will be communicated to investigators and trial registries by the study team (Additional file 6).

### Consent

All participants will provide written informed consent. Consent will be taken by a research nurse or a registered medical professional with suitable study training, as delegated by the PI at each Regional Centre. The process for obtaining participant informed consent will be in accordance with REC guidance and GCP. One copy of the ICF will be kept by the participant, one will be kept by the Investigator, and a third will be retained in the site file at the GP practice; practice staff will be asked to scan this into the patients’ electronic GP record (Additional file 2).

Participants will have received a PIS in advance of their consent visit (at least 24 h), allowing them ample time to consider their participation. The research nurse will explain the details of the trial and will answer any questions that the participant has concerning study participation (Additional file 3).

The decision regarding participation in the study is entirely voluntary. The investigator or their nominee shall emphasise that consent regarding study participation may be withdrawn at any time without penalty or affecting the quality or quantity of their future medical care, or loss of benefits to which the participant is otherwise entitled.

The consent form states that information collected about participants will be used to support other research in the future, and may be shared anonymously with other researchers.

### Confidentiality

Individual participant medical information obtained as a result of this study are considered confidential and disclosure to third parties without consent is prohibited except where required to meet regulatory requirements. All trial staff will adhere to the principles of GCP and the General Data Protection Regulation 2018.

### Access to data

Access to study data will be restricted to relevant study personnel who are aware of the importance of subject confidentiality. Data generated by the study will be analysed by statisticians and health economists at the University of Southampton. The Chief Investigator will have control of and act as custodian for data generated by the study. No biological specimens data are collected for trial purposes, though routinely collected tests results will be part of the trial dataset.

### Ancillary and post-trial care

Insurance and indemnity for trial participants and staff is provided through NHS schemes (under cover of Health Service Guidelines [95] 48) and Public Liability Insurance/Clinical Trials Insurance held by the Sponsor.

### Dissemination policy

#### Trial results

The results of ATTACK will be reported in peer-reviewed journals and scientific meetings. The results will also be disseminated to guideline committees, NHS organisations and patient groups. Patients will be informed of the results of the trial once they have been published via a newsletter or the ATTACK public website.

#### Authorship

This will be determined according to guidelines from the International Committee of Medical Journal Editors. Other contributors will be acknowledged. No use of professional writers is intended.

## Discussion

CKD affects at least 12% of adults and is a powerful risk factor for CVD. Evidence on new approaches to prevent CVD in CKD is urgently required.

There is currently insufficient evidence to recommend the use or avoidance of aspirin for the primary prevention of CVD in CKD as data on the use of antiplatelet agents in the specific setting of primary prevention in CKD are limited. The literature suggests that the efficacy of aspirin in CVD prevention is at least as great in people with CKD as the general population but the risks may also be greater, and so uncertainty remains about the net balance of benefit and risk. In 2014, the National Institute for Health and Care Excellence (NICE) made a research recommendation for a definitive trial of aspirin for primary prevention of CVD in people with CKD.

In the UK, it has been estimated that more than 3 million people with CKD and no pre-existing CVD are not prescribed aspirin and around one million are receiving aspirin in the absence of definitive evidence [97]. The results of this trial, whether positive or negative, will therefore be directly and immediately applicable to very large numbers of patients. ATTACK is the first definitive trial of aspirin as primary CVD prevention in CKD patients. The open design and pragmatic approach to bleeding prophylaxis and low platelet counts mean that the results will accurately reflect the real-world application of aspirin prophylaxis in the UK and hence will be of great interest to clinicians, guideline groups and policy-makers, in the UK and globally, particularly given the high and rising prevalence of CKD. The low cost of aspirin means that a positive result would also be of relevance to low- and middle-income countries and the impact not diluted in countries such as the USA by issues around income or insurance status.

## Trial status

The current approved version of the Protocol is version 4.0, dated 24th September 2021. The first patient consented on 26th February 2019. The trial was paused as a result of the Coronavirus pandemic in March 2020 and restarted, with a suite of modifications designed to render the study fully Covid-secure in March 2021. This paper describes the redesigned trial.

## Supplementary Information

**Additional file 1.** WHO dataset.

**Additional file 2.** Informed Consent Form.

**Additional file 3.** Participant Information Sheet.

**Additional file 4.** Endpoint definitions.

**Additional file 5.** SPIRIT checklist.

**Additional file 6.** Protocol versioning.

## Data Availability

The ATTACK study Protocol will be made publically available on the NIHR website. The Statistical Analysis Plan will be available upon request. Requests for controlled access to the datasets generated and/or analysed during this study will be considered by the Sponsor, taking into consideration all legal and regulatory requirements. Where requests are approved, individual participant data will be shared after de-identification and normalisation of information (text, tables, figures and appendices).

## Abbreviations

ATC: Antithrombotic Trialists’ Collaboration
ATTACK: Aspirin To Target Arterial Events In Chronic Kidney Disease
CI: Chief Investigator
CKD: Chronic kidney disease
CKD-EPI: Chronic Kidney Disease Epidemiology Collaboration
CRF: Case report form
CRN: Clinical Research Networks
CTU: Clinical trials unit
CVD: Cardiovascular disease
DMEC: Data Monitoring and Ethics Committee
EAC: Endpoint Adjudication Committee
eGFR: Estimated glomerular filtration rate
EPR: Electronic patient record
EQ-5D-5L: EuroQol five dimensions (EQ-5D) 5 level
GCP: Good Clinical Practice
GI: Gastrointestinal
HCRW: Health and Care Research Wales
HEAT: *Helicobacter* Eradication Aspirin Trial
HES: Hospital Episode Statistics
HOT: Hypertension Optimal Treatment
HR: Hazard ratio
HRA: Health Research Authority
HRQoL: Health-related quality of life
HTA: Health Technology Assessment
ICD: International Classification of Diseases
ICF: Informed consent form
ICH: International Conference on Harmonisation
ITT: Intention-to-treat
JPAD: Japanese Primary Prevention of Atherosclerosis with Aspirin for Diabetes
KDIGO: Kidney Disease Improving Global Outcomes
MCV: Mean cell volume
MDRD: Modification of Diet in Renal Disease
MHRA: Medicines and Healthcare products Regulatory Agency
MI: Myocardial Infarction
NICE: National Institute for Health and Care Excellence
NIHR: National Institute for Health Research
ONS: Office for National Statistics
OPCS: Office of Population Censuses and Surveys
PCR: Protein:creatinine ratio
PI: Principal Investigator
PIS: Participant Information Sheet
PPI: Proton pump inhibitor
QALY: Quality-adjusted life year
QOF: Quality and Outcomes Framework
REC: Research Ethics Committee
RRID: Renal Risk in Derby
SAE: Serious adverse event
SHARP: Study of Heart and Renal Protection
SOP: Standard Operating Procedures
SUSAR: Suspected Unexpected Serious Adverse Reaction
TIA: Transient ischaemic attack
TSC: Trial Steering Committee
UACR: Urine albumin:creatinine ratio
