# Supplementary material for: Aspirin to target arterial events in chronic kidney disease (ATTACK): study protocol for a multicentre, prospective, randomised, open-label, blinded endpoint, parallel group trial of low-dose aspirin vs. standard care for the primary prevention of cardiovascular disease in people with chronic kidney disease
Source: Trials. 2022 Apr 21;23:331. doi: 10.1186/s13063-022-06132-z (PMC9021558; doi:10.1186/s13063-022-06132-z)
Supplement: Supplementary file 1 — Additional file 1. WHO dataset. [file 13063_2022_6132_MOESM1_ESM.doc]

WHO Trial Registration Data Set

| Primary registry and trial identifying number | EudraCT: 2018-00644-26 |
| --- | --- |
| Date of registration in primary registry | 9^th^ October 2018 |
| Secondary identifying numbers | IRAS Project ID: 228831  Sponsor Reference: 31844  ISRCTN: ISRCTN40920200  ClinicalTrials.gov: NCT03796156  NIHR Reference 16/31/127  British Heart Foundation SP/17/14/33355 |
| Source(s) of monetary or material support | National Institute for Health Research Health Technology Assessment Programme  British Heart Foundation |
| Primary sponsor | University of Southampton |
| Secondary sponsor(s) | None |
| Contact for public queries | Jennifer Dumbleton (Trial Manager) jennifer.dumbleton@nottingham.ac.uk |
| Contact for scientific queries | Hugh Gallagher (Chief Investigator) hugh.gallagher1@nhs.net |
| Public title | ATTACK: Aspirin in Chronic Kidney Disease |
| Scientific title | ATTACK: Aspirin To Target Arterial events in Chronic Kidney disease |
| Country of recruitment | United Kingdom |
| Health condition(s) or problem(s) studied | Chronic kidney disease (stages 1-4) |
| Intervention(s) | Active comparator: aspirin 75 non-enteric coated (plain tablet or dispersible) once daily in addition to routine care  Comparator: usual care and avoidance of aspirin |
| Key inclusion and exclusion criteria | Inclusion  Males and females aged 18 years and over at the date of screening  Subjects with CKD (reduced eGFR and/or albuminuria) defined as:   - estimated glomerular filtration rate [eGFR] <60mL/min/1.73m2 for at least 90 days, and/or - kidney disease code on the GP electronic patient AND most recent eGFR in CKD-defining range (<60mL/min/1.73m2), and/or - albuminuria or proteinuria (defined as urine albumin:creatinine ratio [ACR] ≥3mg/mmol, and/or urine protein:creatinine ratio [PCR] ≥15mg/mmol, and/or +protein or greater on reagent strip)   Subjects willing to give permission for their paper and electronic medical records to be accessed and abstracted by trial investigators for the duration of the trial  Subjects willing to be contacted and interviewed by trial investigators should the need arise for adverse event assessment  Subjects able to communicate well with the investigator or designee, to understand and comply with the requirements of the study and to understand and sign the written informed consent  Subjects who are willing to give permission for their paper and electronic medical records to be accessed by trial investigators  Subjects who are willing to be contacted and interviewed by trial investigators  Subjects who can communicate well with the investigator or designee, understand the requirements of the study and understand and sign the written informed consent |
|  | Exclusion  Subjects with CKD GFR category 5  Subjects with pre-existing cardiovascular disease (angina, myocardial infarction, stroke, transient ischaemic attack (TIA), significant peripheral vascular disease, coronary or peripheral revascularisation for atherosclerotic disease)  Subjects with a current pre-existing condition associated with increased risk of bleeding other than CKD  Subjects currently prescribed anticoagulants or antiplatelet agent, or taking over the counter (OTC) aspirin continuously  Subjects who are currently and regularly taking other drugs with a potentially serious interaction with aspirin  Subjects with a known allergy to aspirin or definite previous clinically important adverse reaction  Subjects with poorly controlled hypertension (systolic blood pressure [BP] ≥180 mmHg and/or diastolic BP ≥105 mmHg)  Other conditions which in the opinion of the GP would preclude prescription of aspirin in routine clinical practice, for example significant anaemia or thrombocytopenia  Subjects who are pregnant or likely to become pregnant during the study period  Subjects with malignancy that is life-threatening or likely to limit prognosis, other life-threatening co-morbidity, or terminal illness  Subjects whose behaviour or lifestyle would render them less likely to comply with study medication  Subjects in prison  Subjects currently participating in another interventional clinical trial or who have taken part in a trial in the last 3 months |
| Study type | Interventional |
|  | Allocation: randomised |
|  | Intervention model: parallel assignment |
|  | Masking: blinded endpoint (outcome assessor) |
|  | Primary purpose: prevention |
|  | Phase IV |
| Date of first enrolment | 26^th^ February 2019 |
| Target sample size | 25,210 |
| Recruitment status | Recruiting |
| Primary outcome(s) | Time to first major vascular event from the date of randomisation. A major vascular event is defined as a primary composite outcome of non-fatal myocardial infarction, non-fatal stroke and cardiovascular death (excluding confirmed intracranial haemorrhage and other fatal cardiovascular haemorrhage). |
| Key secondary outcomes | Efficacy  Death from any cause  Composite outcome of major vascular event or revascularisation (coronary and non-coronary)  Individual components of the primary composite endpoint  Health-related quality of life  Safety  Composite outcome of intracranial haemorrhage (fatal and non-fatal), fatal extracranial haemorrhage and non-fatal major extracranial haemorrhage (adjudicated)  Fatal and non-fatal (reported individually and as a composite) intracranial haemorrhage comprising: i) primary haemorrhagic stroke (to distinguish from haemorrhagic transformation of ischaemic stroke); ii) other intracranial haemorrhage (adjudicated)  Fatal and non-fatal (reported individually and as a composite) major extracranial haemorrhage: i) upper gastrointestinal; ii) lower gastrointestinal; ii) sight-threatening ocular; iv) multiple trauma; v) other (adjudicated)  Clinically relevant non-major bleeding (if hospitalised) (adjudicated)  Composite outcome of fatal and non-fatal major extracranial haemorrhage and clinically relevant non-major bleeding (if hospitalised) |
| Tertiary outcomes | Transient ischaemic attack  Unplanned hospitalisation  Hospitalisation with heart failure  New diagnosis of cancer (colorectal/other)  Death due to cancer (where cancer is the underlying cause of death)  CKD progression  New diagnosis of dementia  Non-traumatic major lower limb amputation |
| Ethics review | The study received approval from the UK Medicines and Healthcare products Regulatory Agency (MHRA) (Reference 16730/0223/001-0001) on 21^st^ September 2018 and a favourable opinion from the East Midlands - Leicester Central Research Ethics Committee (REC) (Reference 18/EM/0248) on 9^th^ October 2018.  Health Research Authority (HRA) and Health and Care Research Wales (HCRW) Approval was granted on 15^th^ October 2018. |
| IPD sharing statement | Participants will be informed that Individual Participant Data (IPD) from ATTACK may be responsibly shared to support efficient clinical research, generate new knowledge and bring benefit to patients. Requests for controlled access to the datasets generated and/or analysed during this study will be considered by the Sponsor, taking into consideration all legal and regulatory requirements. Where requests are approved, IPD will be shared after de-identification and normalisation of information (text, tables, figures, and appendices). |
|  |  |
