# Supplementary material for: Aspirin to target arterial events in chronic kidney disease (ATTACK): study protocol for a multicentre, prospective, randomised, open-label, blinded endpoint, parallel group trial of low-dose aspirin vs. standard care for the primary prevention of cardiovascular disease in people with chronic kidney disease
Source: Trials. 2022 Apr 21;23:331. doi: 10.1186/s13063-022-06132-z (PMC9021558; doi:10.1186/s13063-022-06132-z)
Supplement: Supplementary file 2 — Additional file 2. Informed Consent Form. [file 13063_2022_6132_MOESM2_ESM.pdf]

REGIONAL CENTRE LOGO

UNIVERSITY OF Southampton

ATTACK

# CONSENT FORM

**Title of Study: Aspirin To Target Arterial events in Chronic Kidney disease (ATTACK)**

**IRAS Project ID: 228831** **ERGO Reference: 31844**

**Name of Researcher**: **<<NAME OF PI>>**

**Patient Screening Number:** ☐☐☐☐☐☐ **--** ☐☐☐☐

**PLEASE INITIAL BOXES**

1. I confirm that I have read and understand the information sheet version number <<XXX>> dated <<XXX>> for the above study and have had the opportunity to ask questions. ☐

2. I understand that my participation is voluntary and that I am free to withdraw at any time, without giving any reason, and without my medical care or legal rights being affected. I understand that should I withdraw then the information collected to date may be used in the final analysis if I agree at this time. ☐

3. I understand that periodically, personal identifiable data, such as my NHS Number and date of birth, will be sent securely to NHS Digital or equivalent NHS bodies, who will be able to provide details about any hospital admissions (Hospital Episode Statistics, HES) I experience, or details from the Office for National Statistics (ONS) in the event of my death or if I develop cancer. I understand that data will always be transferred in a secure fashion. I give permission for my data to be used in this way. ☐

4. I understand that relevant sections of my medical notes, data collected in the study and applicable information from the ONS and HES will be uploaded to the study database and looked at by authorised individuals from the University of Southampton, the research team and regulatory authorities where it is relevant to my taking part in this study. I give permission for these individuals to have access to these records for the duration of the study and to collect, store, analyse and publish information obtained from my participation in this study. I understand that my personal details will be kept confidential except for the secure sharing of data with NHS Digital or equivalent NHS bodies. ☐

5. I am happy for disclosure of my identity to the trial coordinating centre for contact (via post, email or telephone) and follow-up purposes. ☐

6. I understand that information collected about me will be used to support other research in the future, and may be shared anonymously with other researchers. ☐

7. I agree to my GP being informed of my participation in this study. ☐

8. I agree to take part in the above study. ☐

| Full Name of Participant (First name, Surname) | Date | Signature |
| --- | --- | --- |
| | | |

File 1 original in trial master file, give 1 original to the participant, file 1 copy in GP Site File
