# Supplementary material for: Aspirin to target arterial events in chronic kidney disease (ATTACK): study protocol for a multicentre, prospective, randomised, open-label, blinded endpoint, parallel group trial of low-dose aspirin vs. standard care for the primary prevention of cardiovascular disease in people with chronic kidney disease
Source: Trials. 2022 Apr 21;23:331. doi: 10.1186/s13063-022-06132-z (PMC9021558; doi:10.1186/s13063-022-06132-z)
Supplement: Supplementary file 3 — Additional file 3. Participant Information Sheet. [file 13063_2022_6132_MOESM3_ESM.pdf]

<<REGIONAL LOGO>>

IRAS Project ID: 228831
ERGO Reference: 31844

PARTICIPANT INFORMATION SHEET

Aspirin To Target Arterial Events In Chronic Kidney Disease

You are invited to take part in a research study

- Before deciding whether to take part, it is important for you to understand why the research is being done and what it will involve.
- Please take time to read this information carefully and discuss it with friends and family if you wish.
- You are free to decide whether or not to take part. If you choose not to take part, this will not affect the care you get from your own doctors, either currently or in the future.
- Taking part involves one consultation ("screening") by telephone or video call with a research nurse to make sure the study is suitable for you. Half of our eligible volunteers will then be allocated at random (by chance) to take a low-dose aspirin daily and half will continue on their usual medications. No face to face or follow-up visits are needed.
- Ask if there is anything that is not clear, or if you would like more information.

Why is this research being done?

- We are doing this research to find out whether people with chronic kidney disease (CKD) should take regular low-dose aspirin to reduce the chance of having a first heart attack or stroke.

What is chronic kidney disease?

- CKD is a term used by healthcare professionals when the kidneys are not working as normal. The word "chronic" means long-term not serious.
- CKD is very common and our kidney function often declines as we get older. Many people are not aware that they have reduced kidney function.
- Most people with chronic kidney disease are treated by their GP and never need to go to hospital to see a kidney specialist.

Why have I been invited?

- You have been invited because tests on your GP record suggest you have chronic kidney disease. This is something you may be aware of or it may be new to you.

Why may I not know I have chronic kidney disease?

- There are usually no symptoms of kidney disease in the early stages. It may only be diagnosed if you have a blood or urine test for another reason (such as for monitoring diabetes or high blood pressure care) and the results show a possible issue with your kidneys.

Part 1 of this information sheet (page 2) explains why we are doing this research and what will happen if you choose to take part.
Part 2 (page 4) gives more detailed information on taking part.

Part 1. Why are we doing the research and what is involved in taking part?

Why are we doing this study?

We are doing this research to find out whether people with chronic kidney disease (CKD) should take daily low-dose aspirin to reduce the risk of a first heart attack or stroke. Currently we believe that in the UK around 1 million people with CKD are already taking aspirin for this reason and around 3 million are not. The results of this study will tell us whether taking aspirin is better for patients.

What is chronic kidney disease?

CKD is a term used by doctors when the kidneys are not working as well as they should. It is very common and affects as many as one in eight adults in the UK. CKD is particularly common in older people and in those with diabetes and high blood pressure.

We diagnose CKD using a blood test (to measure kidney function) and a urine test (to measure if the kidneys are leaking protein). People are often unaware that they have chronic kidney disease unless their doctor mentions it.

We say something is “chronic” when it is present for more than three months, but because urine protein tests are not measured very often you will be eligible to take part in our research even if there is only one abnormal reading.

Why is CKD important?

CKD is important because it is linked to a much higher chance of heart attacks and strokes. On average 2 or 3 of every 100 people with CKD will have a heart attack or stroke every year. The risk of heart attack or stroke in people with mild CKD is double the risk in people with normal kidney function. The risk increases to five times as high as CKD worsens. We therefore need to find ways to reduce these risks.

Using aspirin to prevent heart attacks and strokes

Heart attacks and strokes are usually caused by small blood clots. Aspirin thins the blood. This reduces the chance that clots will form but also leads to an increased risk of bleeding.

Studies in people with **previous** heart attacks or strokes show that aspirin reduces the risk of further attacks, and that these benefits are much greater than the risks of bleeding. As a result, aspirin is recommended for people (both with CKD and without CKD) who have already had a heart attack or stroke.

Aspirin is less beneficial in preventing a **first** attack or stroke in the general population and is generally not recommended for this purpose.

As heart attacks and strokes are far more common in people with CKD than in the general population, we would expect aspirin to be of greater benefit, but the risks may also be higher as bleeding is more common in people with reduced kidney function. Before we can recommend aspirin treatment to help a first heart attack or stroke in people with CKD, we need to be sure that the benefits of treatment outweigh the possible risks.

There is already some evidence suggesting that aspirin is effective in preventing a first heart attack or stroke in people with CKD and that the benefits outweigh the risks, but the evidence is not conclusive. The National Institute of Health and Care Excellence (NICE) therefore recommended that a definitive research study was needed.

The results of our research study will indicate more clearly whether people with chronic kidney disease (CKD) should take daily low-dose aspirin to reduce the risk of a first heart attack or stroke.

Why have I been invited?

We are inviting all potentially suitable people to take part in our study. This includes people aged 18 or over whose GP records and test results indicate they have CKD and who have not previously had a heart attack or a stroke.

If you think you might like to be involved in this study, please read the rest of this information sheet.

Do I have to take part?

No, it is entirely up to you to decide whether you would like to take part in the research study. If you would like to be considered, please return the enclosed reply slip to the research team in the envelope provided, and one of our team will contact you.

If you do take part, you will be free to withdraw from the study at any time without giving a reason. Withdrawal from the study will not affect the standard of care that you receive in any way, either currently or in the future.

What is involved if I decide to take part?

Consent consultation

If you decide to take part, we will contact you by telephone to make **just one appointment** for a consent consultation. This will take place by telephone or video call to avoid the risk of Covid-19 transmission. Face-to-face appointment will only be made if national guidelines allow and this is preferred. A research nurse will discuss the study with you and make sure that the study is suitable for you. You will also be able to ask questions about the study. If you are unsure whether to take part, you can ask to have another consultation or take longer to think about the study before making your decision.

If you are happy to join the study after this discussion, you will be asked to sign a consent form. The consent form confirms that you agree to take part and that you give us permission to look at your computerised medical records (now and in the future) from your GP and from any hospital you attend. Relevant sections of your medical records will be uploaded to the trial database to help with the trial's analysis. We will ask you to post the signed consent form back to us in the pre-paid envelope provided.

After you have signed the consent form, we will ask some questions about your medical history and also ask you to complete a simple questionnaire.

What happens after the consent consultation?

You will be allocated at random (by chance) to:

- take low-dose aspirin (75mg) once daily in addition to your regular prescribed medication (this will be an uncoated tablet or soluble aspirin, which are more effective and more commonly prescribed by GPs than coated tablets)

  OR:

- continue with your regular medication alone.

There is no "dummy pill" (we call this a placebo) so you will know which group you are in. We will also confirm this in writing with you.

If you take part in the study, it is important that you do not take any "over-the-counter" aspirin (i.e. that has not been prescribed by a doctor). This includes cold and flu remedies that contain aspirin.

<u>How will the research team find out whether aspirin is beneficial?</u>

Once the study starts, we will find out whether you have had a heart attack or stroke or experienced any episodes of bleeding by analysing your GP and hospital electronic records. The data collected from your medical record at consent will be updated on a regular basis electronically from your GP practice record.

Once a year we will contact you by letter/email and ask you to complete a basic questionnaire about your health. **<u>You will not be asked to attend any appointments (at the GP surgery or the hospital) as part of the study.</u>**

We plan to include around 25,000 people in the study from across the UK. We expect that the study will continue for about six years, so depending on when you start in the trial you could be prescribed aspirin for between approximately 2.5 and 6 years. At the end of the study, we will have collected information about a large group of people with CKD who have taken aspirin regularly and another group of people with CKD who have not taken aspirin regularly. If we find differences in the rates of heart attack, stroke or bleeding between these two groups we can assess whether these differences are due to the effects of aspirin. This will help us to decide how best to treat people with CKD in the future. When the study is finished, we will let you know the results of the research by letter or email.

What are the possible benefits of taking part?

Until we complete the research, we will not know whether taking aspirin benefits people with CKD. Taking part in the study may not benefit you personally, but the information we get will improve the treatment of people with CKD in the future.

We believe that the results of this trial (whether positive or negative) will be extremely important for the NHS:

- If the benefits of aspirin are shown to outweigh the risks, then treatment will be immediately available to about 3 million people with CKD. We estimate that this will help prevent around 50,000 heart attacks and strokes across the UK over a five-year period.
- If there is no overall benefit, we will have firm evidence that the estimated 1 million people with CKD who are currently on aspirin to reduce the risk of a first heart attack or stroke should stop taking it.

As well as helping patients, we believe that this trial is also important as it may lead to major cost savings for the NHS. This is because aspirin is an inexpensive drug and the costs of heart attack and stroke in people with CKD are very high (up to £1 billion per year).

What are the possible disadvantages?

Aspirin has been used for medical purposes since 1899 and is already recommended and widely used in people with CKD who have had a previous heart attack or stroke. Low dose aspirin is generally safe but like all drugs, aspirin can have side effects. The side effects of aspirin are well known. The most common side effects are indigestion and irritation of the stomach. The most important side effect is bleeding, particularly from the stomach and intestine. Your GP may prescribe an additional medication to reduce stomach irritation.

Previous studies in people with CKD have shown that treatment with aspirin to help prevent heart disease may result in one extra serious bleeding episode per 500 people treated each year.

The most serious possible side-effect is bleeding in the brain. This is rare, with 1-2 additional bleeds for every 10,000 people in the general population taking aspirin to prevent heart disease each year.

Aspirin may have additional risks in certain groups, such as people with asthma, women with heavy periods and anyone undergoing surgery (such as dental surgery) and extra precautions may need to be taken, such as telling your dentist you are taking part in this study. Please speak to the research nurse or your GP if you have any concerns.

Part 2. Further information

Expenses and payments

Participation in this research study is voluntary and you will not be paid for taking part. If you are allocated to the group taking aspirin, this will be prescribed to you by your GP. If you are under 60 and pay prescription charges, these will be reimbursed to you. If your GP chooses to prescribe additional medication to reduce stomach irritation, the prescription charges for this will also be reimbursed. The research nurse will explain the process for this if applicable to you when you speak to them.

Will my taking part in the study be kept confidential?

All information that we collect about you during the study will be kept strictly confidential. The information collected will be stored securely on a password-protected database. Electronic data will be encrypted. Only authorised personnel will have access.

Responsible members of the University of Southampton (and the relevant NHS Trust(s)/ site/ coordinating centre) will use your name, NHS number, date of birth and contact details to contact you about the research study, to make sure that relevant information about the study is recorded for your care, and to oversee the quality of the study. Individuals from the University of Southampton and regulatory organisations may look at your medical and research records to check the accuracy of the research study. The only people in the University of Southampton who will have access to information that identifies you will be people who need to contact you for follow-up or those auditing the data collection process. The people who analyse the information will not be able to identify you and will not be able to find out your details. All of these people have a duty to keep your information, as a research participant, strictly confidential.

Whilst you are taking part in the study, we will also request health related information about you from NHS Digital, the Department of Health body responsible for NHS Information Technology. Periodically, personal identifiable data, such as your unique NHS identifier (your “NHS number”) and date of birth, will be sent securely to NHS Digital and we will obtain permission for them to provide further details about you. This may include, for example, details of any hospital stay or any other clinical event, such as a new diagnosis of cancer. Any information about you that leaves the hospital will have your name and address removed. A unique code will be used so that you cannot be directly identified from it.

Data Protection Information / GDPR

The University of Southampton is the Sponsor for this study based in the UK. We will be using information from you and your medical records to undertake this study and will act as the data controller for this study. This means that we are responsible for looking after your information and using it properly in a fair, lawful and secure manner. The University of Southampton will securely store your information for up to 10 years after the study has finished, after which time any link between you and your information will be removed.

Your rights to access, change, or move your information are limited, as we need to manage your information in specific ways to comply with legislation governing the conduct of research studies and to ensure the research output is reliable and accurate. If you withdraw from the study, we will keep the information about you that we have already obtained. To safeguard your rights, we will use the minimum personally identifiable information possible.

Data will always be transferred in a secure NHS-approved fashion. TCR Nottingham is a data processor of your data, responsible for the design, management and security of the study database (http://www.tcrnottingham.com/Privacy/TCR Privacy Notice.pdf). The study team will be responsible for managing access to your data.

Making patient data from clinical trials available to other researchers in the future is regarded as good practice and offers many well-recognised advantages that can advance research and benefit patients. Requests from other researchers for controlled access to data generated in this study will be considered by the Sponsor, taking into consideration all legal and regulatory

requirements. Where requests are approved individual patient data will be shared with other researchers in a way that makes sure they cannot identify you.

You can find out more about how we use your information by contacting us (see details at the end of this document).

What if new information becomes available?

Sometimes during a trial, new information becomes available about the treatment that is being studied. If this happens, the research team will write to you to ask if you wish to continue in the study. If you decide to continue, we may ask you to sign an updated consent form.
We will let you know if the study is stopped for any reason and explain what the reason is.

What happens if I don't want to carry on with the study?

Your participation is voluntary and you are free to withdraw at any time. You do not have to give any reason and your legal rights will not be affected.

What if there is a problem?

If you have a concern about any aspect of this study, please speak to the researchers who will do their best to answer your questions. Their contact details are given at the end of this information sheet. If you remain unhappy or have a complaint about any aspect of this study, please contact the Research Integrity and Governance Manager at University of Southampton (023 8059 5058, rgoinfo@soton.ac.uk). You can also raise your complaint through the NHS Complaints Procedure. Details can be obtained from your GP or your local Patient Advice and Liaison Service (PALS) Office (https://www.nhs.uk/service-search/other-services/Patient-advice-and-liaison-services-(PALS)/LocationSearch/363, or telephone NHS111 and request details of your local PALS Office).

What will happen to any samples I give?

We will not be collecting any samples for this study.

What will happen to the results of the research study?

After the study has finished, we will publish the results in scientific journals and present them at scientific meetings. Your details will remain strictly confidential and you will not be directly identifiable by name from any such report or publication. We will also send a summary of the results to patient groups, all participants and their GP.

It is highly likely that the findings of the research will influence future national and international guidelines on the care of patients with CKD.

Who is organising and funding the research?

The study has been organised by a team of researchers from different UK Universities, led by the University of Southampton. It is being funded by the National Institute for Health Research (NIHR) Health Technology Assessment (HTA) programme and the British Heart Foundation.

Who has reviewed the study?

To protect your interests, all research in the NHS is looked at by an independent group of people called a Research Ethics Committee. This study has been reviewed and approved by East Midlands – Leicester Central Research Ethics Committee.

Further details

If you would like to see a short video describing the study, please go to <<INSERT URL HERE>>, There are also further details on our study website: <<INSERT URL HERE>>.

How to contact us

If you have any questions or would like more information about the study please contact your Regional Centre: <<*Regional centre contact details*>>

Thank you for taking the time to read this information sheet.
