# Supplementary material for: Aspirin to target arterial events in chronic kidney disease (ATTACK): study protocol for a multicentre, prospective, randomised, open-label, blinded endpoint, parallel group trial of low-dose aspirin vs. standard care for the primary prevention of cardiovascular disease in people with chronic kidney disease
Source: Trials. 2022 Apr 21;23:331. doi: 10.1186/s13063-022-06132-z (PMC9021558; doi:10.1186/s13063-022-06132-z)
Supplement: Supplementary file 4 — Additional file 4. Endpoint definitions. [file 13063_2022_6132_MOESM4_ESM.docx]

**Endpoint definitions**

| **Major vascular event** | Composite outcome of non-fatal myocardial infarction, non-fatal stroke and cardiovascular death (excluding confirmed intracranial and other cardiovascular haemorrhage) |
| --- | --- |
| **Non-fatal myocardial infarction** | Defined according to the Fourth Universal Definition of MI (1). This is included in full below for reference but it should be emphasised that the information required for unambiguous event ascertainment according to these definitions is unlikely to be available to adjudicators.  Note:   - Primary adjudication outcome will be MI or not MI. It is recognised that distinguishing between Type 1 and Type 2 MI on the basis of the information available will be challenging. Where the discharge summary strongly implies a Type 2 MI (for example documented severe anaemia or atrial fibrillation with a minor troponin rise) the event will be recorded as Type 2; otherwise all will be labeled as Type 1. The primary outcome measure will include all MI within the composite, with a secondary analysis reporting on Type 1 MI only. - It is likely that in many cases it will be unclear whether biomarker criteria for acute MI described below are met. In these examples the disease codes and narrative from the discharge summary should be used to reach a judgement on whether an MI has taken place; source data will not be made available - As in the ASCEND trial (2), prior/silent MI is excluded - Codes indicating non-MI chest pain will be logged but not formally adjudicated. This will also allow ascertainment of some out of hospital deaths.   The following definitions are included for reference:  *Criteria for myocardial injury*  The term myocardial injury should be used when there is evidence of elevated cardiac troponin values (cTn) with at least 1 value above the 99th percentile upper reference limit (URL). The myocardial injury is considered acute if there is a rise and/or fall of cTn values.  *Criteria for acute myocardial infarction (Types 1, 2 and 3 MI)*  The term acute myocardial infarction should be used when there is acute myocardial injury with clinical evidence of acute myocardial ischemia and with detection of a rise and/or fall of cTn values with at least 1 value above the 99th percentile URL and at least 1 of the following:   - Symptoms of myocardial ischaemia; - New ischemic ECG changes; - Development of pathological Q waves; - Imaging evidence of new loss of viable myocardium or new regional wall motion abnormality in a pattern consistent with an ischaemic aetiology; - Identification of a coronary thrombus by angiography or autopsy (not for types 2 or 3 MIs).   Post-mortem demonstration of acute atherothrombosis in the artery supplying the infarcted myocardium meets criteria for Type 1 MI.  Evidence of an imbalance between myocardial oxygen supply and demand unrelated to acute atherothrombosis meets criteria for Type 2 MI.  Cardiac death in patients with symptoms suggestive of myocardial ischaemia and presumed new ischaemic ECG changes before cTn values become available or abnormal meets criteria for Type 3 MI.  *Criteria for coronary procedure–related myocardial infarction (Types 4 and 5 MI)*  Percutaneous coronary intervention (PCI)–related MI is termed Type 4a MI.  Coronary artery bypass grafting (CABG)–related MI is termed Type 5 MI.  Coronary procedure-related MI ≤48 hours after the index procedure is arbitrarily defined by an elevation of cTn values >5 times for Type 4a MI and >10 times for Type 5 MI of the 99th percentile URL in patients with normal baseline values. Patients with elevated pre-procedural cTn values, in whom the pre-procedural cTn level are stable (≤20% variation or falling), must meet the criteria for a >5 or >10 fold increase and manifest a change from the baseline value of >20%. In addition with at least 1 of the following:   - New ischaemic ECG changes (this criterion is related to Type 4a MI only) - Development of new pathological Q waves - Imaging evidence of loss of viable myocardium that is presumed to be new and in a pattern consistent with an ischaemic aetiology - Angiographic findings consistent with a procedural flow-limiting complication such as coronary dissection, occlusion of a major epicardial artery or graft side-branch occlusion-thrombus, disruption of collateral flow or distal embolisation   Isolated development of new pathological Q waves meets the Type 4a MI or Type 5 MI criteria with either revascularisation procedure if cTn values are elevated and rising but less than the pre-specified thresholds for PCI and CABG.  Other types of 4 MI include Type 4b MI stent thrombosis and Type 4c MI restenosis that both meet Type 1 MI criteria.  Post-mortem demonstration of a procedure-related thrombus meets the Type 4a MI criteria or Type 4b MI criteria if associated with a stent.  *ECG criteria consistent with myocardial ischaemia*   - ST elevation. New ST elevation at the J point in two contiguous leads with the cut-points: ≥0.1 mV in all leads other than leads V2-V3 where the following cut-points apply: ≥0.2 mV in men ≥40 years (≥0.25 mV in men <40 years) or ≥0.15 mV in women regardless of age   ST depression and T-wave changes. New horizontal or down-sloping ST *depression* ≥ 0.5 mm in two contiguous leads and/or new T inversion >1 mm in two contiguous leads with prominent R wave or R/S ratio >1  Lesser ECG abnormalities may represent an ischaemic response. In patients with known or high likelihood of coronary artery disease, the clinical presentation is critical to enhance the specificity of these findings.  *Myocardial injury/infarction associated with CKD*   - Many patients with CKD have raised cTn - Diagnosing MI in patients with CKD and elevated cTn may be difficult if symptoms or ECG changes absent - However serial changes in cTn are equally effective in diagnosing MI in those with and without CKD - If a rising or falling pattern is present the aetiology of the abnormal cTn values could be acute volume overload, congestive heart failure or MI. If a rising or falling pattern is accompanied by ischaemic symptoms, new ischaemic ECG changes or loss of viable myocardium on imaging, a diagnosis of acute MI is likely |
| **Non-fatal stroke** | This is defined in accordance with the World Health Organization (WHO) definition as “rapidly developing clinical signs of focal (or global) disturbance of cerebral function, with symptoms lasting 24 hours or longer, with no apparent cause other than of vascular origin” (3). This excludes cases of primary cerebral tumour, cerebral metastasis, subdural haematoma, post-seizure palsy, brain trauma and TIA. Haemorrhagic stroke (fatal and non-fatal) which has been confirmed on appropriate imaging (see below) is excluded from the primary composite endpoint and included within the secondary endpoints. Haemorrhagic transformation of a primary ischaemic stroke is included within the primary endpoint. Haemorrhagic stroke includes both intracerebral and subarachnoid haemorrhage (4,5). Uncertain stroke will be categorised as ischaemic.  As with myocardial infarction, adjudicators will be strongly encouraged to reach an adjudication on the basis of available information. Where this information is insufficient to distinguish between ischaemic and haemorrhagic stroke, source data may be requested. |
| **Cardiovascular death** | Defined largely according to the work of the Standardised Data Collection for Clinical Trials Initiative (6,7) . Note:   - Deaths due to intracranial haemorrhage (haemorrhagic stroke, non-stroke intracranial haemorrhage) are, as safety events rather than efficacy targets, excluded from the primary composite endpoint and included within the secondary endpoints - Other elements of death due to cardiovascular haemorrhage (for example non-procedural or non-traumatic vascular rupture, or haemorrhage causing cardiac tamponade) are also defined as safety events and including within the secondary endpoints - Deaths due to vascular trauma leading to fatal haemorrhage (eg knife injuries) will be categorised as deaths due to trauma (non-cardiovascular) - Aim of adjudication is to capture the primary cause of death, defined as the underlying disease that initiated the chain of events resulting in death (as opposed to the mode of death which is the physiological derangement or biochemical disturbance produced by the cause of death). Non-cardiovascular causes of death may culminate in a cardiovascular mode of death (from example renal failure cause dysrhythmia) – these will not be regarded as CV deaths.   Cardiovascular death is subdivided as follow:   - Death due to acute MI, defined as one of:   - Death by any cardiovascular mechanism ≤30 days after a MI (definite or suspected)   - Death resulting from a procedure to treat a MI or a complication resulting from MI   - Cardiac death with symptoms suggestive of myocardial ischaemia and presumed new ischaemic ECG changes or LBBB, but death occurred before cardiac biomarkers were obtained, or before cardiac biomarker values would be increased - Sudden cardiac death is a death that occurs unexpectedly, not following an acute MI, and includes the following deaths:   - Death witnessed and occurring without new or worsening symptoms   - Death witnessed within 60 minutes of the onset of new or worsening cardiac symptoms, unless the symptoms suggest acute MI   - Death witnessed and attributed to an identified arrhythmia (e.g. captured on an ECG recording, witnessed on a monitor, or unwitnessed but found on implantable cardioverter-defibrillator review)   - Death after unsuccessful resuscitation from cardiac arrest (e.g. implantable cardioverter defibrillator unresponsive sudden cardiac death, pulseless electrical activity arrest)   - Death after successful resuscitation from cardiac arrest and without identification of a specific cardiac or non-cardiac aetiology   - Unwitnessed death in a subject seen alive and clinically stable ≤24 hours prior to being found dead without any evidence supporting a specific non-cardiovascular cause of death - Death due to stroke (excluding confirmed haemorrhagic stroke), where the death is either a direct consequence of the stroke or a complication of the stroke - Death due to heart failure, where the death is in association with clinically worsening symptoms and/or signs of heart failure regardless of HF aetiology - Death due to cardiovascular procedures, where the death is caused by the immediate complications of a cardiac procedure (excluding deaths from procedures to treat an MI) - Death due to other cardiovascular causes is a cardiovascular death not included in the above categories (and not due to intracranial or cardiovascular haemorrhage) but with a specific, known cause (e.g. pulmonary embolism or peripheral arterial disease)   The definitions for fatal stroke and MI will be operationalised as follows:  *Coronary heart disease*   - Death on same calendar day as myocardial infarct will be classified as fatal MI (unless there is clear evidence for alternative cause of death) - Death up to 30 days after definite/probable MI which is due to any cardiovascular mechanism OR where the cause of death is unknown will be classified as fatal MI (single event) - Death up to 30 days after MI where the cause of death (other than MI) can be determined/deduced will be classified as non-fatal MI followed by non-MI death event (two events) - Death from any cause more than 30 days after MI will be classified as non-fatal MI followed by death event (two events)   *Stroke*   - Death up to 30 days after stroke regardless of severity will be classified as fatal stroke (single event) unless there is evidence for an undeniable other cause of death (eg MI, malignancy, accidental) (which will be classified as non-fatal stroke followed by non-stroke death event) - Death more than 30 days after severe stroke (value judgement; equivalent to modified Rankin Score >3) will be classified as fatal stroke (single event) unless there is evidence for an undeniable other cause of death, eg MI, malignancy, accidental (which will be classified as non-fatal stroke followed by non-stroke death event) - Death more than 30 days after non-severe stroke (equivalent to modified Rankin Score 3 or less) will be classified as death due to other cause (non-fatal stoke followed by non-stroke death) |
| **Haemorrhagic stroke** | Defined in accordance with the World Health Organization (WHO) definition of stroke as “rapidly developing clinical signs of focal (or global) disturbance of cerebral function, with symptoms lasting 24 hours or longer or leading to death, with no apparent cause other than of vascular origin” (3), and including both intracerebral haemorrhage and subarachnoid haemorrhage.  Events will be adjudicated pragmatically. Where source data is available, the approach of the ASPREE investigators will be followed to confirm the diagnosis:   - CT scanning demonstrates an area of hyperdensity within the brain parenchyma with or without extension into the ventricles or subarachnoid space or, for scans performed beyond 1 week, an area of attenuation with ring enhancement after injection of contrast, or - MRI scanning shows an area of hypointensity or isointensity on T1-weighted images or an area of marked hypointensity on gradient echo and T2-weighted images, or - autopsy demonstrates the origin of the hemorrhage as the cerebral parenchyma (8)   For reporting purposes primary intracerebral haemorrhage and subarachnoid haemorrhage will be grouped and recorded as haemorrhagic stroke. |
| **Intracranial haemorrhage** | Includes intracerebral haemorrhage, subarachnoid haemorrhage, subdural haemorrhage, and epidural haemorrhage. For reporting purposes, subdural and epidural haemorrhage will be grouped and recorded as other intracranial haemorrhage. Intracranial bleeding will be further sub-classified as traumatic and non-traumatic (9). |
| **Major extracranial haemorrhage** | Major extracranial bleeding is defined as:   - Fatal bleeding, or - Symptomatic bleeding in a critical area or organ, such as intraspinal, intraocular, retroperitoneal, intra-articular or pericardial, or intramuscular with compartment syndrome, or - Bleeding that leads to the transfusion of two or more units of whole blood or red cells   Major bleeds are those that result in death, are life-threatening, cause chronic sequelae or consume major health-care resources. In particular, to be classified as major, bleeds in a critical area or organ should:   - Be associated with a symptomatic clinical presentation (not following an incidental finding) - Be the cause of the symptoms   The definitions follow the recommendations of the International Society for Thrombosis and Haemostasis (ISTH) (10), with the difference that “bleeding causing a fall in hemoglobin level of 20 g/L or more” is included within the ISTH definition but excluded in ATTACK. Change in haemoglobin has been removed because patients with CKD may have, or develop, anaemia as a direct result of the kidney disease, and may be treated with erythropoietin which will result in fluctuations in the haemoglobin concentration, making the relationship between bleeding and haemoglobin level less clear. A change in haemoglobin was also excluded from their definition of “clinically significant bleeding” by the ASPREE investigators (9).  It is recognised that in many cases the information available from the primary data source will be insufficient to determine beyond doubt whether the criteria for major bleeding have been met. In such cases surrogate markers of major bleeding reported in the discharge summary will also be acceptable:   - Bleeding with reported or implied haemodynamic compromise - Bleeding requiring urgent surgery or angiography for haemostasis - GI bleeding for which urgent inpatient endoscopy is arranged   The following decision rules, adapted from ASPREE (9), will also be followed:   - Bleeding following elective inpatient surgery or endoscopic procedures is not counted as major bleeding (even if other markers of severity such as transfusion are evident) - Readmission for bleeding after elective surgical or endoscopic procedure, or admission after elective outpatient surgical or endoscopic procedure will be counted as major bleeding if any other markers of severity present - Bleeding following non-elective inpatient surgical or endoscopic procedure will be counted as major bleeding if any other markers of severity present   Unlike in ASPREE or ASCEND, hospitalisation or prolongation of hospitalisation per se are not criteria for major bleeding. This follows the approach of the ISTH, justified “because bleeding is extremely unlikely to be the primary cause of serious medical consequences without also satisfying one of the included criteria for major bleeding, and inclusion of this criterion has the potential to falsely classify a minor bleed as a major bleed because of the coincidental occurrence of other conditions (10)”.  The source of major bleeding will be categorised as upper gastrointestinal, lower gastrointestinal, sight threatening ocular, multiple trauma and other.  It is recognised that ocular bleeds may present a particular challenge as many will not be admitted to hospital. GP codes strongly suggestive of sight threatening eye bleeds will be used to identify possible events; the EAC coordinator will obtain and redact additional information from the GP for the committee to review. |
| **Clinically relevant non-major bleeding** | Defined in accordance with the ISTH as any sign or symptom of haemorrhage (e.g. more bleeding than would be expected for a clinical circumstance, including bleeding found by imaging alone) that does not fit the criteria for the ISTH definition of major bleeding but does meet at least one of the following criteria:   - Requiring medical intervention by a healthcare professional - Leading to hospitalisation or increased level of care - Prompting a face to face (i.e. not just a telephone or electronic communication) evaluation (11)   This definition includes all minor bleeding episodes that lead to medical evaluation involving direct patient contact.  Ascertainment of CRNBM in an open trial will be subject to bias. We will therefore confine our reporting of CRNMB to that leading to hospitalisation or occurring as a hospital inpatient, as this will be formally adjudicated.  CRNMB outside hospital will be logged from coded GP events but not formally reported. |
| **Cause of death** | Aim of adjudication is to capture the primary cause of death, defined as the underlying disease that initiated the chain of events resulting in death (as opposed to the mode of death which is the physiological derangement or biochemical disturbance produced by the cause of death).  Will be ascertained from death certificates and post-mortem reports, and classified as:   - Cardiovascular death (within the primary endpoint, ie excluding intracranial haemorrhage and other cardiovascular haemorrhage) - Death due to haemorrhagic stroke - Death due to other intracranial haemorrhage - Death due to extracranial haemorrhage - Death due to cancer - Other non-cardiovascular death (a definitive non-cardiovascular/non-haemorrhagic cause of death must be identified) - Undetermined cause of death.   A common analytic approach for cause of death analyses is to assume that all undetermined cases are included in the CV category (e.g. presumed CV death, specifically “death due to other CV causes”) (7). This approach will be followed in ATTACK, and a sensitivity analysis performed with these deaths as non-cardiovascular. |
| **Revascularisation** | Will include open and percutaneous coronary and non-coronary (including carotid, aortic and limb) procedures (as defined in OPCS-4 procedure codes) and will be ascertained from HES data. |
| **Notes on selected non-adjudicated events** | **TIA** will be ascertained from HES data and also from GP coded data as many TIA will be treated outside hospital. TIA events will not be formally adjudicated.  **Hospitalisation** defined as an official admission that is for a duration greater than 24 hours or a minimum of 2 calendar days where exact time of stay is unavailable. HES will serve as primary data source (not adjudicated).  **Admissions for heart failure** will be derived from HES data.  **Cancer registrations** will be derived from HES/ONS.  **CKD progression** defined as at least one of:   - >30% fall in eGFR over two years (12) , or - need for renal replacement therapy or 50% decline in eGFR (13) **,** or - new eGFR<15mL/min/1.73m^2^, or - 25% decline in GFR together with a drop in GFR category (14)   **New diagnoses of dementia** will be taken from GP coded data.  **Major lower limb amputation** is defined as the surgical removal of a part or whole limb proximal to the ankle. Will be ascertained from HES (not adjudicated).  **Health-related quality of life (HRQoL**) is a combination of a person’s physical, mental and social well-being, not merely the absence of disease. A 'utility' is the measure of the preference or value that an individual or society gives a particular health state. It is generally a number between 0 (representing death) and 1 (perfect health). The most widely used measure of benefit in cost-utility analysis is the quality-adjusted life year (QALY), which combines quality of life with length of life. One QALY is equal to one year of life in perfect health. QALYs are calculated by estimating the years of life remaining for a patient following a particular treatment or intervention and weighting each year with a quality of life score (on a 0 to 1 scale). It can be measured in terms of the person’s ability to carry out the activities of daily life, and freedom from pain and mental disturbance (15). |

1. Thygesen K, Jaffe AS, Chaitman BR, Canada PJD. Fourth Universal De fi nition of Myocardial Infarction ( 2018 ). J Am Coll Cardiol [Internet]. 2018; Available from: https://doi.org/10.1016/j.jacc.2018.08.1038

2. The ASCEND Study Collaborative Group. Effects of Aspirin for Primary Prevention in Persons with Diabetes Mellitus. New Engl J Med. 2018;379:1529–39.

3. The World Health Organization MONICA Project (monitoring trends and determinants in cardiovascular disease): a major international collaboration. WHO MONICA Project Principal Investigators. J Clin Epidemiol [Internet]. 1988;41(2):105–14. Available from: http://www.ncbi.nlm.nih.gov/pubmed/3335877

4. Johnston SC, Amarenco P, Albers GW, Denison H, Easton JD, Evans SR, et al. Ticagrelor versus Aspirin in Acute Stroke or Transient Ischemic Attack. N Engl J Med [Internet]. 2016;375(1):35–43. Available from: http://www.nejm.org/doi/10.1056/NEJMoa1603060

5. Eikelboom JW, Connolly SJ, Bosch J, Dagenais GR, Hart RG, Shestakovska O, et al. Rivaroxaban with or without Aspirin in Stable Cardiovascular Disease. N Engl J Med [Internet]. 2017;NEJMoa1709118. Available from: http://www.nejm.org/doi/10.1056/NEJMoa1709118

6. Hicks KA, Hung HMJ, Mahaffey KW, Mehran R, Nissen SE, Stockbridge NL, et al. Standardized Definitions for Cardiovascular and Stroke End Point Events in Clinical Trials. 2014;1–33.

7. Hicks KA, Mahaffey KW, Meran R, Nissen SE. 2017 Cardiovascular and Stroke Endpoint Definitions for Clinical Trials. Circulation. 2018;137:961–72.

8. ASPREE Protocol Version 9 November 2014.

9. Margolis KL, Mahady SE, Nelson MR, Ives DG, Satterfield S, Britt C, et al. Development of a standardized definition for clinically significant bleeding in the ASPirin in Reducing Events in the Elderly (ASPREE) trial. Contemp Clin Trials Commun. 2018;11(May):30–6.

10. Schulman S, Kearon C. Definition of major bleeding in clinical investigations of antihemostatic medicinal products in non-surgical patients. J Thromb Haemost. 2005;3(4):692–4.

11. Kaatz S, Ahmad D, Spyropoulos AC, Schulman S. Definition of clinically relevant non-major bleeding in studies of anticoagulants in atrial fibrillation and venous thromboembolic disease in non-surgical patients: Communication from the SSC of the ISTH. J Thromb Haemost. 2015;13(11):2119–26.

12. Coresh J, Turin TC, Matsushita K, Sang Y, Ballew SH, Appel LJ, et al. Decline in estimated glomerular filtration rate and subsequent risk of end-stage renal disease and mortality. JAMA [Internet]. 2014 Jun 25;311(24):2518–31. Available from: http://www.ncbi.nlm.nih.gov/pubmed/24892770

13. Feldman HI, Appel LJ, Chertow GM, Cifelli D, Cizman B, Daugirdas J, et al. The Chronic Renal Insufficiency Cohort (CRIC) Study: Design and Methods. J Am Soc Nephrol [Internet]. 2003 Jul;14(7 Suppl 2):S148-53. Available from: http://www.ncbi.nlm.nih.gov/pubmed/12819321

14. KDIGO. KDIGO 2012 Clinical Practice Guideline for the Evaluation and Management of Chronic Kidney Disease. Kidney Int Suppl [Internet]. 2013;3(1):4–4. Available from: http://www.kdigo.org/clinical_practice_guidelines/pdf/CKD/KDIGO CKD-MBD GL KI Suppl 113.pdf%5Cnhttp://www.nature.com/doifinder/10.1038/kisup.2012.73%5Cnhttp://www.nature.com/doifinder/10.1038/kisup.2012.76

15. Glossary | NICE. [cited 2017 Dec 21]; Available from: https://www.nice.org.uk/glossary
