# Supplementary material for: Aspirin to target arterial events in chronic kidney disease (ATTACK): study protocol for a multicentre, prospective, randomised, open-label, blinded endpoint, parallel group trial of low-dose aspirin vs. standard care for the primary prevention of cardiovascular disease in people with chronic kidney disease
Source: Trials. 2022 Apr 21;23:331. doi: 10.1186/s13063-022-06132-z (PMC9021558; doi:10.1186/s13063-022-06132-z)
Supplement: Supplementary file 6 — Additional file 6. Protocol versioning. [file 13063_2022_6132_MOESM6_ESM.docx]

Appendix – Protocol version 4.0

Issue date: 24-Sep-21

Author(s):

Revision Chronology

| Amendment No. | Protocol Version No. | Date issued | Author(s) of changes | Details of changes made |
| --- | --- | --- | --- | --- |
|  |  |  |  |  |
| Ethics meeting response | 4.0 | 1-Feb-22 |  |  |
| Substantial amendment 7 | 4.0 |  |  | An addendum to the Participant Information Sheet for participants recruited in Wales, to clarify the processes for routine data collection in this region |
| MHRA response | 4.0 | 9-Nov-21 |  |  |
| Ethics meeting response | 4.0 | 18-Oct-21 |  |  |
| Substantial amendment 6 | 4.0 |  |  | Protocol amended to include some broadened inclusion criteria, and to clarify some of the exclusion criteria |
| MHRA response | 3.1 | 19-Nov-20 |  |  |
| Ethics meeting response | 3.1 | 17-Nov-20 |  |  |
| Substantial amendment 5 | 3.1 |  |  | Changes to make the trial remote, in light of the Covid-19 pandemic |
| Ethics meeting response | 2.0 | 11-Jun-20 |  |  |
| MHRA response | 2.0 | 5-Jun-20 |  |  |
| Substantial amendment 4 | 2.0 |  |  | Review of protocol and update of accompanying documentation in light of - recently published trials of aspirin in other populations, operational experiences (for example: how proteinuria is reported by laboratories; potential for external events to influence timelines), discussions with endpoint adjudication committee chairs |
| Ethics meeting response | 1.1 | N/A |  |  |
| MHRA response | 1.1 | 24-Mar-20 |  |  |
| Substantial amendment 3 | 1.1 |  |  | Temporary halt due to Covid-19 |
| Ethics meeting response |  | 8-Dec-19 |  |  |
| Substantial amendment 2 | 1.1 |  |  | Addition of new recruitment areas |
| Ethics meeting response | 1.1 | 9-Oct-18 |  |  |
| MHRA response | 1.1 | 21-Sep-18 |  |  |
| Substantial amendment 1 | 1.1 |  |  | Addition of study website |
| …………………………………… |  |  |  |  |
